# Triboelectric Mat Multimodal Sensing System (TMMSS) Enhanced by Infrared Image Perception for Sleep and Emotion‐Relevant Activity Monitoring

**DOI:** 10.1002/advs.202407888

**Published:** 2024-12-19

**Authors:** Jinlong Xu, Xinge Guo, Zixuan Zhang, Huajun Liu, Chengkuo Lee

**Affiliations:** ^1^ Department of Electrical and Computer Engineering National University of Singapore Singapore 117583 Singapore; ^2^ Center for Intelligent Sensors and MEMS (CISM) National University of Singapore Singapore 117608 Singapore; ^3^ Institute of Materials Research and Engineering (IMRE) Agency for Science Technology and Research (A*STAR) Singapore 138634 Republic of Singapore; ^4^ National University of Singapore Suzhou Research Institute (NUSRI) Suzhou Industrial Park Suzhou 215123 China; ^5^ NUS Graduate School‐Integrative Sciences and Engineering Programme (ISEP) National University of Singapore Singapore 119077 Singapore

**Keywords:** deep learning, digital twin, multimodality, smart home, triboelectric sensor

## Abstract

To implement digital‐twin smart home applications, the mat sensing system based on triboelectric sensors is commonly used for gait information collection from daily activities. Yet traditional mat sensing systems often miss upper body motions and fail to adequately project these into the virtual realm, limiting their specific application scenarios. Herein, triboelectric mat multimodal sensing system is designed, enhanced with a commercial infrared imaging sensor, to capture diverse sensory information for sleep and emotion‐relevant activity monitoring without compromising privacy. This system generates pixel‐based area ratio mappings across the entire mat array, solely based on the integral operation of triboelectric outputs. Additionally, it utilizes multimodal sensory intelligence and deep‐learning analytics to detect different sleeping postures and monitor comprehensive sleep behaviors and emotional states associated with daily activities. These behaviors are projected into the metaverse, enhancing virtual interactions. This multimodal sensing system, cost‐effective and non‐intrusive, serves as a functional interface for diverse digital‐twin smart home applications such as healthcare, sports monitoring, and security.

## Graphical abstract

The triboelectric mat multimodal sensing system, enhanced with a commercial infrared imaging sensor, can capture triboelectric information and thermal images simultaneously without privacy concerns. Additionally, it utilizes multimodal sensory intelligence and deep‐learning analytics to detect different sleeping postures and monitor comprehensive sleep behaviors and emotional states associated with daily activities.

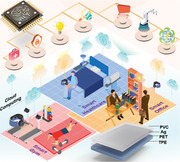

## 1 Introduction

With the rapid advancements in the Internet of Things (IoT) and artificial intelligence (AI), the deployment of sensory nodes has become widespread, allowing for real‐time detection and seamless communication of sensory information.^[^ ^1^, ^2^, ^3^, ^4^, ^5^ ^]^ This progress supports the integration of numerous functional sensors with cloud computing, aiding the development of comprehensive systems such as robotic perception,^[^ ^6^, ^7^ ^]^ health monitoring,^[^ ^8^, ^9^, ^10^ ^]^ and smart homes.^[^ ^11^ ^]^ Additionally, it extends to specialized applications like plant monitoring,^[^ ^12^ ^]^ medical delivery,^[^ ^13^, ^14^, ^15^ ^]^ smart retail,^[^ ^16^ ^]^ and smart traffic management.^[^ ^17^, ^18^ ^]^ The concept of a digital twin plays a pivotal role in this technological landscape by using interactive sensing data to replicate physical systems virtually. Alongside, significant advancements include the metaverse, which merges virtual reality (VR) and augmented reality (AR) technologies to facilitate multisensory interactions between virtual environments and the physical world.^[^ ^19^, ^20^, ^21^ ^]^ Powered by AI, digital twins analyze diverse sensory data from wearable electronics and ambient devices,^[^ ^22^, ^23^ ^]^ effectively bridging the physical and virtual worlds within the metaverse.^[^ ^24^, ^25^, ^26^, ^27^, ^28^, ^29^ ^]^ Wearable electronics, known for their mechanical flexibility and stretchability, conform to the skin or can be implanted,^[^ ^30^, ^31^, ^32^, ^33^, ^34^ ^]^ precisely monitoring physical, chemical, and biological signals.^[^ ^35^, ^36^, ^37^, ^38^, ^39^ ^]^ Meanwhile, ambient sensors, unconstrained by scalability or weight, are installed to gather extensive environmental information such as temperature, humidity, light levels, and occasionally sounds or motions.^[^ ^40^, ^41^ ^]^

Recently, the integration of AI with IoT, known as AIoT, has emerged as a cutting‐edge technology for various intelligent systems.^[^ ^42^, ^43^, ^44^, ^45^ ^]^ To build more robust and intelligent systems, mainstream microelectromechanical system (MEMS) sensors, prized for their compact sizes and low power consumption, are increasingly used.^[^ ^45^, ^46^, ^47^ ^]^ However, their reliance on external power supplies can limit mobility and flexibility and increase system complexity.^[^ ^48^, ^49^, ^50^ ^]^ Addressing these challenges, piezoelectric nanogenerators (PENGs) and triboelectric nanogenerators (TENGs) harness self‐generated signals from external mechanical stimuli for self‐powered sensing are developed.^[^ ^51^, ^52^, ^53^, ^54^ ^]^ The TENGs work based on the triboelectric effect, a coupling effect of contact electrification and electrostatic induction using two different materials.^[^ ^55^, ^56^, ^57^ ^]^ Compared to PENGs, TENGs offer more advantages in material compatibility, cost, and flexibility, making them ideal for various applications.^[^ ^58^, ^59^, ^60^, ^61^ ^]^

In terms of digital‐twin smart home applications, visible imaging sensors combined with advanced image processing algorithms are commonly used for human motion recognition and location detection. However, Video recordings may reveal sensitive personal details such as facial features, compromising users’ privacy. As an alternative, a low‐cost, low‐power triboelectric mat monitoring system, which also supports large‐area fabrication, offers a promising solution without compromising privacy.^[^ ^62^, ^63^ ^]^ In 2020, Ma et al. introduced a flame‐retardant smart carpet for precise position detection and optimal escape route guidance.^[^ ^64^ ^]^ Yet, the limited machine learning capabilities of the system restrict its functionality in smart buildings and other applications. In the same year, Shi et al. enhanced the system by integrating self‐powered floor sensors connected in parallel to reduce complexity and by introducing deep learning (DL) algorithms for improved sensing of stepping positions, activities, and identities.^[^ ^65^ ^]^ Despite these advances, the long‐term stability of TENG sensors is compromised by high humidity sensitivity, an issue Shi et al. addressed by developing humidity‐resistant triboelectric coding mats.^[^ ^66^ ^]^ However, the common one‐step‐one‐pixel method for walking trajectory recognition remains underutilized in practical applications, requiring further refinement. By 2023, Yang et al. had developed a robust two‐channel TENG Infomat system for arbitrary position sensing, which was particularly effective in yoga training within digital‐twin smart homes.^[^ ^19^ ^]^ Yet, this system primarily detects gait information, potentially overlooking critical details like upper body movements, which could limit its usefulness in analyzing complex activities in the metaverse.^[^ ^67^ ^]^

Meanwhile, smart mats can also function as mattresses, enhancing healthcare monitoring by facilitating the observation of sleep conditions.^[^ ^68^, ^69^, ^70^, ^71^ ^]^ Unlike traditional wearables monitoring physiological signals,^[^ ^72^, ^73^, ^74^, ^75^, ^76^ ^]^ triboelectric mat arrays significantly reduce body discomfort during prolonged use. In 2018, Lin et al. proposed large‐scale washable textiles based on TENG technology for real‐time monitoring of sleep positions and evaluating sleep quality.^[^ ^77^ ^]^ In 2022, Kou et al. introduced a flexible and breathable TENG sensor array designed as a pillow.^[^ ^78^ ^]^ This self‐powered pillow monitors head positions and movement trajectories and includes an early warning system for fall hazards. Although the TENG monitoring system can detect sleep positions, its lack of machine‐learning algorithms might limit the comprehensiveness of the sleep analysis reports generated. Capabilities such as individual identification, action recognition, location detection, and preliminary sleep monitoring have been achieved by analyzing signals from the TENG system.^[^ ^79^, ^80^ ^]^

Although the TENG monitoring system already provides valuable data, there are still opportunities to enhance its functionality for monitoring sleep and digital‐twin interactions for complex activities such as emotion‐related motions. By integrating additional sensors, the system can achieve multimodal functionalities, which allow it to capture a broader range of information on sleep postures and daily activities.^[^ ^81^, ^82^, ^83^ ^]^ Particularly, in the smart home sensing regime, the functionality of the TENG sensing system can be effectively improved through an imaging sensor.^[^ ^84^, ^85^, ^86^ ^]^ However, while standard visible image sensors may pose privacy issues due to their complex image data formats and detailed facial information, infrared imaging offers an unobtrusive and energy‐saving solution. With its simplified data structures and no need for external lighting, infrared imaging sensors offer the same functionality as visible light sensors while enhancing privacy by discreetly detecting and visualizing infrared radiation emitted by objects. This capability makes them particularly suitable for sleep monitoring under smart home scenarios where minimal intrusion and low‐light environments are essential.^[^ ^87^, ^88^ ^]^ Therefore, integrating a triboelectric mat array with an infrared imaging sensor, which can effectively capture thermal images of sleep postures and upper body motions,^[^ ^89^ ^]^ is a promising direction. This integration allows for effective data fusion, potentially yielding high classification accuracy, and alleviates privacy concerns by providing hardware‐level protection of user privacy, thereby avoiding privacy issues that may arise from post‐processing. Such a combination of TENG mats with an infrared imaging sensor would create a highly efficient triboelectric smart home monitoring system, although no such design has been reported.

**Figure 1:**
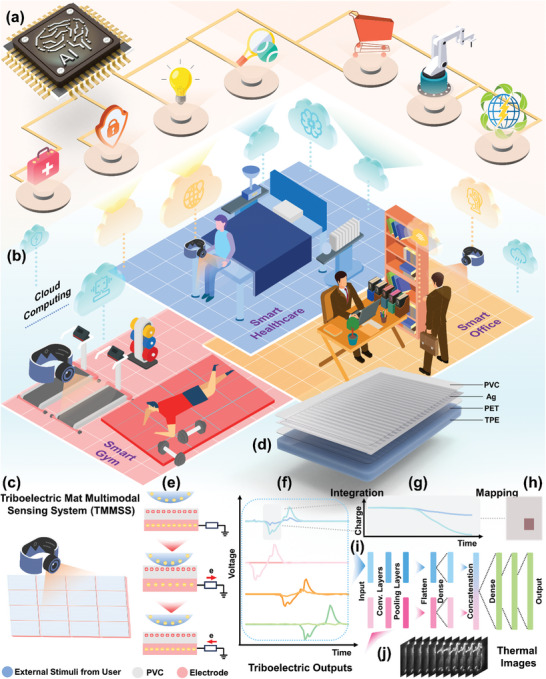
An overview of the triboelectric mat multimodal sensing system (TMMSS) enabled by the integration of a triboelectric mat array and a commercial infrared imaging sensor. a) The schematic of TMMSS equipped with artificial intelligence for various potential applications. b) The illustration of TMMSS layout in some scenarios (smart healthcare, smart gym, and smart office) in smart homes. c) Schematic of TMMSS equipped with the multimodal infrared and triboelectric mat sensors exhibiting unobtrusive cognitive capability for various smart home applications. d) The detailed structure diagram of one triboelectric mat pixel with its precise dimension in Figure S3 (Supporting Information). e) Working principles of triboelectric mat sensor implementation: touch drives and free electron flow. f) The robust triboelectric outputs (Figure S1, Supporting Information) are directly generated by the triboelectric mat array. g) The integral values of the triboelectric voltage. h) The corresponding pixel‐based area ratio mapping by triboelectric data. i) The multimodal convolutional neural network (CNN) for triboelectric and thermal data analysis. j) The thermal images captured by an infrared imaging sensor when the user is on the bed.

Herein, we design a Triboelectric Mat Multimodal Sensing System (TMMSS) featuring a triboelectric mat array alongside an infrared imaging sensor for various applications in areas like smart factories, and smart homes (**Figure** **1**a). The TMMSS, integrated with advanced multimodal AI algorithms, opens the door for more immersive and interactive digital‐twin smart home applications and the metaverse (Figure 1b). In the system (Figure 1c), the triboelectric mat array is composed of a universal design mat pixel, where the interdigital electrodes (IDEs) have a width ratio of 8:2, approximating the comparative areas of the two electrodes, shown in Figure 1d. The working principle of the mat sensor is illustrated in Figure 1e. When a user touches the triboelectric layer, the difference in electrical potential between the electrode and the ground causes free electrons to move from the ground to the electrode, generating a current in the circuit. When the user leaves the sensing pixel being touched, electrons return to the ground, producing an opposite triboelectric current until a new electrical equilibrium is established. 4 distinct mat types have been developed by employing various wiring strategies, each producing robust triboelectric outputs even in high‐humidity environments (Figure S1, Supporting Information), as demonstrated in Figure 1f. These mats are configured into a 4 × 4 array with 8 outputs to optimize the trade‐off between classification accuracy and system complexity. This array can further monitor sleep positions using a voltage integral computation method and generate pixel‐based area ratio mappings (Figure 1g,h). An infrared imaging sensor above the mat array captures thermal images for unobtrusive sleep monitoring and daily behavior recognition (Figure 1j). When the user interacts with the system, triboelectric information generated by the mat matrix and infrared image data captured by the infrared imaging sensor are simultaneously produced and subsequently sent to the multimodal CNN network (Figure 1i) for the recognition of sleep posture (Figure S2, Supporting Information) or a simple assessment of emotional states. For sleep posture detection, this fusion at the score level achieves an impressive classification accuracy of 96.67% across three users and four sleeping positions, surpassing the accuracy from thermal or triboelectric information alone. This system provides a comprehensive sleep monitoring interface that determines the duration of various sleep postures in each stage without compromising privacy. It also measures standard deviations in time‐average area ratio mappings from triboelectric outputs to indicate body movement intensity during each sleep cycle. Additionally, it facilitates advanced digital‐twin interactions in the smart home, particularly for recognizing emotion‐related activities. By employing data fusion analytics, daily activities, whether emotional or non‐emotional, are accurately recognized and projected into the virtual space. Compared to the state‐of‐the‐art works for smart mats or mattresses listed in Table S1 (Supporting Information), the TMMSS opens the door to more immersive and interactive digital‐twin applications. Therefore, the TMMSS with unobtrusive multimodal recognition for sleep monitoring and emotional‐relevant behaviors shows great potential for various digital‐twin smart home applications in the metaverse.

## 2 Design and Characterization of Triboelectric Mat Array for Position Monitoring and Area Mapping

**Figure 2:**
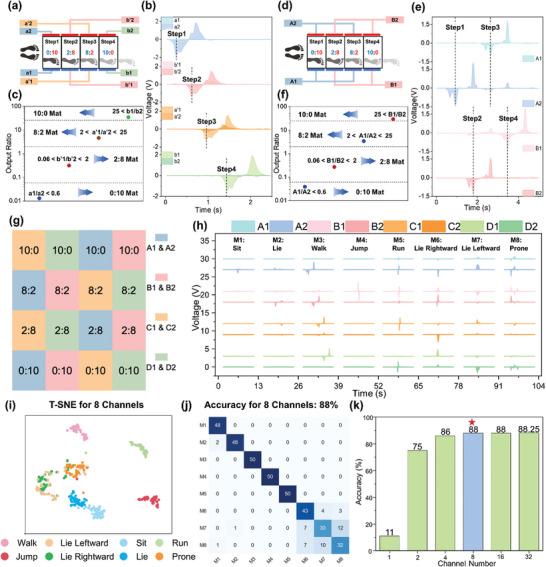
Investigation of the electrode connection of triboelectric mat for position sensing and action recognition. a) The schematic diagram of the separate connection of 4 mats into 8 electrodes. b,c) The generated output voltages and corresponding negative peak‐to‐peak voltage ratio with 4 steps. Subsequently, the classification of different mat pixels is achieved by calculating the voltage ratio and comparing it with the predetermined voltage ratio threshold (Figure S4, Supporting Information). d) The schematic diagram of the interval parallel connection of 4 mats into 4 electrodes. e,f) The generated output voltages and corresponding negative peak‐to‐peak voltage ratio with 4 steps. Subsequently, the identification of the mat pixels with different voltage ratios is similarly achieved using the pre‐calculated voltage ratio thresholds (Figure S4, Supporting Information). g) The schematic diagram of the constructed 4 × 4 pixels‐mat array with 8 output channels (detailed wiring configurations in Figure S5d, Supporting Information). The practical photo of the mat array is illustrated in Figure S6 (Supporting Information). h) Voltage output for different motions (sit, lie, walk, jump, run, lie rightward, lie leftward, and prone) generated by mat array. i) The t‐SNE results of the TENG signal from 8 channels’ mat array. j) Confusion map of recognizing 8 motions for 8 channels’ mat array using a 4‐layer CNN structure (Figure S7, Supporting Information). k) The bar graph illustrating the accuracy of recognizing 8 different motions using a 4×4 pixel matrix with various output channel wiring strategies (the detailed wiring connections in Figure S5, Supporting Information). The corresponding confusion maps are depicted in Figure S8 (Supporting Information).

To scale mat implementation for smart home applications, reducing output ports is crucial for system simplification. The interval connection method for electrodes (E1, E2) efficiently reduces the number of terminals without sacrificing data integrity, as illustrated by the comparison between Figure 2a–f. The precise dimension of the electrode design is illustrated in Figure S3 (Supporting Information). **Figure** **2**a illustrates the schematic diagram of a mat array using 4 mats with different voltage ratios (E1:E2), which are not interconnected with each other. This configuration includes 4 sets of electrodes: a1 and a2, a'1 and a″2, b″1 and b″2, and b1 and b2, depicted in blue, orange, pink, and green, respectively. In the wiring configurations shown in Figure S4a (Supporting Information), the voltage ratios for the 4‐pixel sets are as follows: set a1 and a2 has a voltage ratio of 0:10, set a″1 and a'2 has a ratio of 2:8, set b'1 and b'2 has a ratio of 8:2, and set b1 and b2 has a ratio of 10:0.During the user's constant one‐step‐one‐pixel walking mode with shoes equipped with polytetrafluoroethylene (PTFE) soles, the 8 channels' generated triboelectric signals are displayed in Figure 2b. Since PTFE has a stronger electron affinity than the PET surface of the mat, stepping on and off the grounded mat can generate a voltage output that first shows a negative voltage followed by a positive voltage. It is well‐known that the number of electrons flowing in and out is equal and proportional to the contact area. Thus, mats with different voltage ratios produce proportional voltage outputs. Figure 2b shows that when the first step is taken, the output at a1 is nearly zero, while the negative peak at a2 reaches almost −2 V, indicating that the first step is on a mat with a 0:10 area ratio. Then, the second step is taken, and the first foot leaves the initial mat. The approximate negative peak at b'2 is −1.25 V and about −0.25 V at b″1, conforming to the 2:8 voltage ratio design. Simultaneously, a2 produces a positive peak to ensure the electrical neutrality of the first mat is maintained. When the third step is taken onto the mat array, the negative peaks at a″2 and a'1 are approximately −1 V and −0.25 V, respectively, indicating a voltage ratio of about 8:2 and suggesting that the third mat is being stepped on. Although the absolute output voltage is slightly lower than that of the 2:8 mat b'1 and b″2, the voltage ratio still approximately matches the preset electrode area ratio. The subsequent positive voltage indicates that the foot has fully left the 8:2 mat. Finally, b1 generates a negative peak output of about −1.5 V, while b2 shows no output, indicating a step on a 10:0 mat. The corresponding voltage ratios of the negative voltage peaks for each of the four steps are plotted in Figure 2c. By applying predefined thresholds (Figure S4b–e and Note S1, Supporting Information), the location of each step can be readily differentiated and determined. This demonstrates the practical utility of voltage ratios in partitioning position judgments, and the corresponding stability tests are discussed in Figure S1a,b (Supporting Information). While this design can accurately achieve position monitoring, using every electrode as an output port leads to excessive output ports in large‐scale applications, thus increasing system complexity. To mitigate this issue, an interval connection method is employed, depicted in Figure 2d. Specifically, the a″2 and a2 ports are consolidated into a single output port, A2. Similarly, a1 and a″1, b″2 and b2, and b'1 and b1 are combined to form output ports A1, B2, and B1, respectively. Figure 2e illustrates that when the user takes the first step, only A2 shows a negative peak of approximately −1.5 V, while A1 shows almost zero output, indicating that the first step was on the 0:10 mat. Subsequently, the corresponding positive output from A2 and the negative voltages from B1 and B2, occurring almost simultaneously, suggest that the user has left the first mat and stepped onto the second mat. The negative peaks of B1 and B2 are approximately −1.2 V and −0.3 V, respectively, with a ratio close to 2:8, indicating that the second step is located on the 2:8 mat. This pattern continues with subsequent steps, revealing the user's walking path across the mats with voltage ratios of 0:10, 2:8, 8:2, and 10:0. Employing the predefined thresholds, the corresponding negative peak voltage ratios perfectly determine the user's position, as shown in Figure 2f. Using the interval connection method, the number of output ports can be halved without affecting the system position monitoring capabilities, thereby significantly reducing system complexity. The detailed summary of characters and abbreviations can be referenced in Tables S2 and S3 (Supporting Information).

Based on the aforementioned study, the interval‐connection method (Figure 2d) has been adopted to design a 4 × 4 minimalist mat array configuration in Figure 2g with its detailed wiring configuration in Figure S5d (Supporting Information). The triboelectric mat array features 4 electrode pairs (A1 and A2, B1 and B2, C1 and C2, and D1 and D2), 8 output terminals in total. Each electrode pair, marked with the same color, connects 4 mats, distinguished by its unique voltage ratio. A photo showing the actual mat array can be found in Figure S6 (Supporting Information). Then triboelectric signals (Figure 2h) are collected during the motion activities (sit, walk, jump, and run) as well as sleeping postures (lie, lie rightward, lie leftward, and prone), providing insights into the contact locations and the intensity of activities. To characterize the differentiability of the triboelectric outputs, for Motion 1(M1), only the A2 serial output generates a blunt negative curve, indicating the user is sitting on the mat with a ratio A1:A2 = 0:10. In the case of Motion 4 (M4), only B1 shows an even pronounced output, characterized by one positive curve and one following negative curve. To investigate the motion, the user first separates the mat with B1:B2 = 10:0 and then contacts the mat through vigorous action, meaning that he is jumping. Furthermore, the Motion 3 (M3) curve distinctly outlines the contact locations and intensity. It is obvious that from the sequence of the temporal events that the user first contacts the mat with A1:A2 = 0:10, followed by the mat with B1:B2 = 2:8, then the mat with C1:C2 = 8:2, and finally the mat with D1:D2 = 10:0. The less sharp voltage outputs suggest a walking activity. Motion 5 (M5) displays significantly sharper voltage outputs, indicating a more intense gait pattern differentiating running from walking. Therefore, various activities can be effectively differentiated solely based on the output from the triboelectric mat array. The t‐distributed stochastic neighbor embedding (t‐SNE) technique is usually utilized for dimensionality reduction, well‐suited for visualizing complex high‐dimensional data in a lower‐dimensional space(typically 2D or 3D). The functionality of t‐SNE plots is to reveal the structure and patterns in the data, such as clustering or grouping, by preserving the relative distances and similarities between data points. Figure 2i illustrates the t‐SNE results of 8 motions from 8‐channel mat array by projecting into two dimensions for visualization. It shows that the performance of feature clustering is good, with sparse motion category overlaps before going through the CNN network. For each motion, data collection involves acquiring 300 data points per output channel, accumulating 2400 points across all eight channels. Repeating each motion 250 times allows for creating training, validation, and testing datasets distributed as 60%, 20%, and 20% of the total data. Figure 2j demonstrates that using a 4‐layer CNN structure (Figure S7, Supporting Information), the classification accuracy for outputs from 8 channels is 88%, which is effective for daily activity detection. If the number of output channels (the detailed wiring connections in Figure S5, Supporting Information) is altered, corresponding classification accuracies and confusion maps are depicted in Figures 2k and S8 (Supporting Information). Although the 32‐channel mat array layout slightly increases the recognition accuracy from 88% to 88.25%, the employed wiring strategy might inevitably cause system complexity and even worse in large‐scale implementation. However, if the number of serial outputs becomes less than 8, the classification accuracy significantly decreases. While reducing the output channels can lower the complexity of the system, it inevitably causes the signals to overlap. The resulting voltage overlaps for multiple activities, especially sleep postures, simultaneously contacting two or more mats with the same output channels. This phenomenon affects the system performance to distinguish daily activities, reducing its discrimination capability. Thus, the 8‐channel mat array emerges as an optimal solution, balancing maximal accuracy and a simple design.

**Figure 3:**
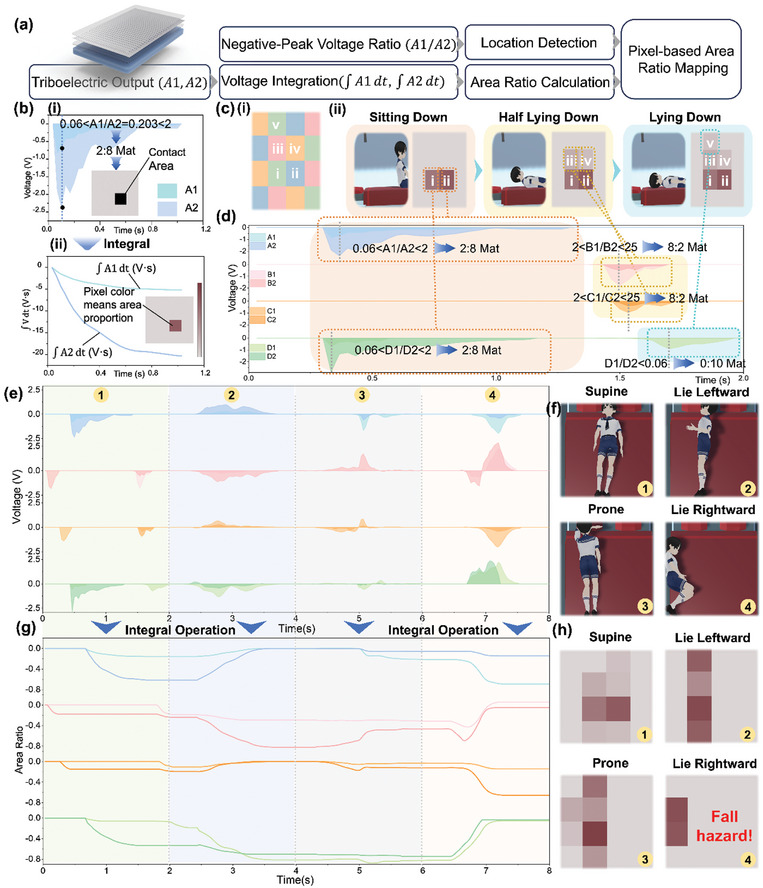
The illustration of triboelectric mat array for sleep position monitoring and area mapping. a) Computational approaches to achieve pixel‐based area ratio mapping. b) The explanation of the principle for calculating the voltage integral in area‐proportional mapping, using a mat pixel with a voltage ratio of 2:8. As illustrated in Figure S9 (Supporting Information) and detailed in Note S2 (Supporting Information), the integrated negative voltage when the contact remains on the entire mat is calculated to be Γ_min_ = −33 V·s, indicating a direct proportionality to the charge transfer. We then use different colors to represent the distinctions between various area ratios, which correspond to the voltage integration ratio. c,d) Progressive demonstration of a man lying down and corresponding outputs and area‐ratio mapping using the same methodology. e,f) The triboelectric outputs of 4 kinds of sleep postures (supine, lie leftward, prone, and lie rightward) and the corresponding demonstration of human actions. g,h) The area ratio of 4 kinds of sleep postures and the corresponding demonstration of area‐ratio mapping diagrams. For the first sleeping posture (supine), the voltage ratio mapping can be directly calculated using the voltage ratio threshold. For the remaining three sleeping postures, since the user presses on two mats with the same output port simultaneously, the voltage ratio mapping can be obtained through matrix operations based on the calculation method described in Note S3 (Supporting Information).

Besides the accurate motion recognition capability, the 4 × 4 mat array can also capture real‐time sleep positions without privacy concerns. In this regard, the computational method of voltage integral is utilized for more in‐depth analysis and discrimination of resting position using TENG data. **Figure** **3**a summarizes how the pixel‐based area ratio mapping is computed. This functionality primarily integrates two sub‐functions: 1) determining the contact area by integrating the triboelectric output from each mat section, and 2) as previously mentioned, identifying the specific mat section being compressed by analyzing the peak‐to‐peak voltage ratio of the negative triboelectric voltage, thereby enabling position detection. In Figure 3bi, the triboelectric outputs A1 and A2 generated when a user contacts the proposed mat array are displayed. Based on the previous analysis, by calculating the negative peak‐to‐peak voltage ratio and referencing the threshold analysis from Figure S4e (Supporting Information), it is determined that the user is contacting a mat section with an A1/A2 = 2:8. Consequently, the corresponding voltage integrals are computed as ∫A1dt and ∫A2dt in Figure 3bii. The contact area Λ and the corresponding integrated voltage output Γ are denoted. It is well known that, in a contact‐separation triboelectric mode, the integral of the voltage, which represents the transferred charge, is proportional to the contact area. When the contact covers the entire surface area of the triboelectric mat, the contact area equals the area of a single mat, denoted as Λ_max_, and the corresponding integrated voltage output is referred to as Γ_min_. From Figure S9 and Note S2 (Supporting Information), it is computed that the integrated negative voltage corresponding to contact without leaving the entire mat is Γ_min_ = −33 V·s, which is directly proportional to the charge transfer. Therefore, based on the above discussion, it is straightforward to establish the relationship between the contact area ratio and the voltage ratio:

(1) $$\frac{\wedge }{\wedge \max}=\frac{\Gamma }{\Gamma \min}$$

Utilizing these voltage integrals, the relationship between contact area and voltage integration can be derived:

(2) $$\frac{\Lambda }{\Lambda \max}=\frac{\Gamma }{\Gamma \min}=\frac{\left(\int A1dt+\int A2dt\right)}{\Gamma \min}\;$$

This procedure is on the premise that the combined surface areas of two IDTs are equivalent to the total area of one mat. For visual clarity, varying colors to represent distinctions are employed between different area ratios, wherein a transition from darker to lighter shades signifies a shift from an area ratio of 1–0.

Figure 3b demonstrates the application of the methodology described in Figure 3a using the example of a person gradually lying down on the 8‐channeled mat array (Figure 3b(i)) with varying sleep positions (Figure 3bii). Corresponding voltage outputs are shown in Figure 3c. During the motion of lying down, initially, A1 and A2, as well as D1 and D2, simultaneously generate a relatively large negative voltage output. Based on the voltage ratio threshold, it can be determined that this action occurs on the mats with A1:A2 = 2:8 and D1:D2 = 2:8. Meanwhile, the absolute value of the corresponding voltage integral is relatively large, indicating that this action occupies a large contact area. Subsequently, B1 and B2, as well as C1 and C2, produce a relatively smaller negative voltage output simultaneously. From the voltage ratio, it is evident that this action occurs on the mats with B1:B2 = 8:2 and C1:C2 = 8:2, and the smaller absolute value of the corresponding voltage integral indicates a smaller contact area for this action. Finally, only the D1 output generates a small voltage output, showing that this action occurs on the mat with D1:D2 = 10:0, and the contact area is also small. Based on the analysis, the variation in pixel‐based area ratio mapping can be calculated as the man moves from a seated position on mats to a fully reclined position, culminating in their head contacting the triboelectric mat array in Figure 3bii. This demonstrates that the triboelectric mat array can comprehensively monitor users' real‐time sleep positions while ensuring the privacy of individuals.

As shown in Figure 3d,e, the user is lying on the mat array following the supine, lie leftward, prone, and rightward sequence and the corresponding real‐time TENG outputs. Furthermore, the integral values for 4 sleep positions and the corresponding area ratio diagram are illustrated in Figure 3f. When the user performs a supine posture, it is observed that electrodes B2 and C2 initially exhibit two small negative peaks. Subsequently, A1, A2, D1, and D2 produce larger and smoother curves, and finally, B1, B2, C1, and C2 successively exhibit smaller negative peaks. This indicates that the person first steps onto the mat with their feet, gradually lying down with their body, and eventually resting their head on the mat. Since the user is not simultaneously pressing on two mats with the same output electrodes, it is possible to determine which mat section is being pressed based on the corresponding electrode pair voltage ratios. This allows for calculating the corresponding area ratio mapping, as illustrated in Figure 3b. However, during the second sleep posture when the user is in a left‐lying position, the voltage output shows positive voltages for electrode pairs A and C, while B and D generate negative voltages. Unlike the output from the first sleep posture, the voltages do not follow the 4 kinds of voltage ratio rules (0:10, 2:8, 8:2, and 10:0). This indicates that the user is simultaneously pressing on two mats with the same output electrodes, resulting in overlapping voltages. In such cases, it is necessary to apply the inherent voltage ratios of the mat design combined with voltage integration for calculation, as detailed in Note S3 (Supporting Information). As the user continues to roll to the left into a prone position and subsequently into a right‐side sleeping position, the corresponding voltage outputs of the third and fourth sleep postures that the electrode pair voltages do not adhere to the 4 kinds of voltage ratio. This means the same method used in the second sleep posture is required to calculate the area ratio mapping. The final voltage ratio integration results are shown in Figure 3g. This real‐time sleep position area ratio mapping can be utilized in home sleep monitoring for the elderly as an automatic fall detection alarm system. When a large area ratio is detected near the boundary of the array, an alarm will be triggered.

Therefore, the 4 × 4 8‐channel triboelectric mat array is designed to detect user position information and classify user behavior while reducing system complexity. Subsequently, by using the voltage integration method, the area ratio mapping of the triboelectric array is achieved to recognize sleeping postures without infringing on user privacy. These functionalities can later be applied to the detection of gait information for complex activities associated with daily life and advanced sleep comprehensive assessment in digital‐twin smart home applications.

## 3 TMMSS with Multimodal Sensory Intelligence and Data Fusion Analytics for Sleep Posture Estimation

**Figure 4:**
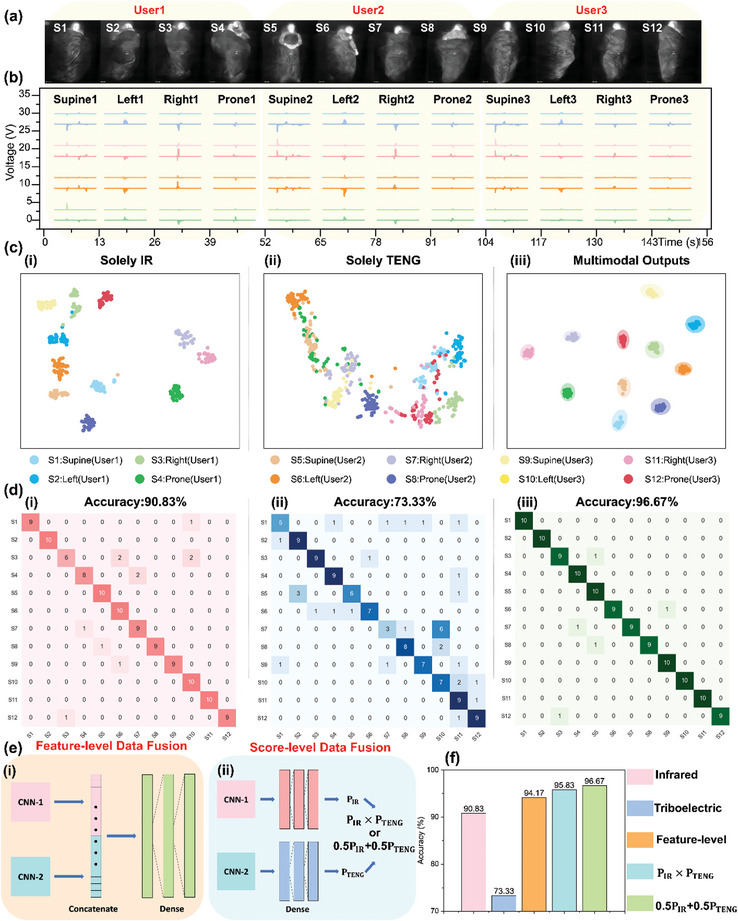
The TMMSS with sensory intelligence and data fusion analytics for sleep posture estimation under thick bedcovers. a) Thermal images for 4 sleep postures of 3 users captured by a commercial infrared imaging sensor for sleep posture estimation without privacy concerns. b) Corresponding triboelectric outputs generated by the triboelectric mat array. c) t‐SNE diagrams of i) thermal data only, ii) triboelectric information only, and iii) multimodal data. d) Sleep posture recognition results of i) infrared data only (detailed network structure in Figure S10, Supporting Information), ii) triboelectric data only (detailed network structure in Figure S11, Supporting Information), and iii) multimodal data. e) The simplified architecture of the multimodal network (detailed multimodal CNN structure for data fusion in Figure S12, Supporting Information) for i) feature‐level and ii) score‐level fusion of the thermal and triboelectric information. f) The recognition performance comparison of thermal data only, triboelectric information only, feature‐level fusion, and score‐level fusion. The confusion maps of feature‐level and weighted score‐level data fusion are illustrated in Figure S13 (Supporting Information).

Though the triboelectric system can detect human activity and the distribution of contact area in a low‐cost, self‐powered, and non‐invasive manner, it still faces some limitations of constrained sensing elements of area ratio mapping and insufficient distinguishability of some sleep postures, such as supine and prone. Advanced DL analytics and integration with other sensors are effective solutions to further enhance the functionality of the triboelectric mat array. With advances in thermal imaging technology, integrating a triboelectric mat array and an infrared imaging sensor is feasible, enhancing sleep posture recognition efficiency and avoiding privacy concerns. An infrared imaging sensor can detect and quantify the thermal radiation emitted by objects, converting it into an electrical signal, which is then processed to generate a visual temperature distribution. The thermal data is collected at a uniform elevation and consistent room temperature while a user rests on the triboelectric mat array. As is shown in **Figure** **4**a, 4‐sleep postures (supine, prone, sleep left‐side, sleep right‐side) of 3 users (User1, User2, and User3) are captured by commercial infrared imaging sensor (FLIR ONE) for sleep posture classification using advanced DL analytics. Unlike digital images, thermal images do not contain facial information, making them suitable for sleep posture recognition without privacy concerns. Thermal data includes information about the user's head position, direction, and upper body movements. The thermal images also reveal inherent identification information, such as body size and hairstyle, since the temperature of hair differs from that of the skin surface. However, it can be seen that under a thick cover, part of the radiation emitted by the user's main body might be shielded, causing limited signals from parts of the body below the upper limbs. Simultaneously, as shown in Figure 4b, the corresponding triboelectric outputs are collected through TENG mats for the 4 sleep postures of 3 users. This data contains information about human movement intensity and position. From the 8‐channel triboelectric data, it is evident that similar outputs for the same posture across different users imply similar coverage positions, while differences in amplitude reflect user identity information.

To characterize the clustering information, the corresponding t‐SNE mapping is shown in Figure 4c. The thermal data is transformed into a 2D format using t‐SNE diagrams, as illustrated in Figure 4ci, to visually demonstrate the capability of the infrared imaging sensor to distinguish sleep postures for different users. The thermal images are capable of classifying different labels, thus detecting the user's resting postures. For each sleep posture, thermal data is captured 50 times per user. Of this data, 80% is allocated for the training process and the remaining 20% for testing. Utilizing a CNN based on thermal data, a recognition accuracy of 90.83% for three users and four sleep postures is achievable, as shown in Figure 4di and the detailed network structure in Figure S10 (Supporting Information). However, some classification errors occur due to the obstruction of thermal radiation emission by thick bedcovers, affecting the accuracy of body position recognition, such as “prone2” and “right1”. Meanwhile, the clustering information of triboelectric outputs is shown in Figure 4cii. It can be seen that the triboelectric information mapping has significant overlap, resulting in weaker classification ability. Using the CNN classification model (Figure S11, Supporting Information), a classification accuracy of 73.33% is achievable, as shown in Figure 4dii. Using these data with a CNN model resulted in a classification accuracy of only 73.33%, indicating that relying solely on the triboelectric array to detect different sleeping postures is insufficient. Some misjudgments, such as “right1” and “right2,” “prone1” and “prone2”, still occur. This is because different users, while in the same sleeping posture, exhibit roughly the same coverage area, which makes it difficult to capture user identity information based solely on triboelectric signals.

Based on the previous discussion, data sourced from the infrared imaging sensor and triboelectric sensors both contribute essential characteristics for monitoring sleep postures. Specifically, thermal data predominantly enhances head directions and upper body motions, while triboelectric data is more concentrated on the movement of the main body. While infrared imaging achieves higher classification accuracy compared with triboelectric mat array, it still faces challenges in lacking the capability to track major body movements beneath thick bedcover. To mitigate these problems and further refine the classification process, employing a multimodal fusion technique presents a viable solution. This approach aims to establish a more robust multimodal perception system by utilizing the complementary effects of different sensors. Therefore, the TMMSS with multimodal perception is designed to achieve higher accuracy in sleep posture recognition.

The multimodal t‐SNE mapping in Figure 4ciii displays outstanding clustering, with most data points distinctly categorized. A multimodal CNN structure for data fusion is depicted in Figure S12 (Supporting Information) to further investigate the classification capability of the multimodal system. Given the disparity in the data formats of thermal images and triboelectric information, multiple convolutional and max‐pooling layers are implemented to create a 1024‐dimension vector for each CNN. As shown in Figure 4ei, following the feature extraction from CNN‐1 and CNN‐2, these 1024‐dimension vectors are concatenated, resulting in a 2048‐dimension vector that serves as the input for a dense network. The classification accuracy, as depicted in Figure S13a (Supporting Information), stands at 94.17%, marginally surpassing the accuracy achieved through the exclusive use of an infrared imaging sensor. The unobvious rise in accuracy is likely due to the intrinsic differences in data types, which usually challenge their integration at feature‐level data fusion. The other method in multimodal data analytics is score‐level data fusion, which allows for the independent processing of each data type, potentially leading to good performance in TMMSS. Figure 4eii demonstrates two potentially prevalent score‐level fusion techniques: weighted average and multiplication. In these methods, P_TENG_ and P_IR_ represent the probability scores for each category from the infrared network (CNN‐1) and the triboelectric network (CNN‐2), respectively. The recognition results of the two methods are shown in Figure 4diii and Figure S13b (Supporting Information). Among all the recognition methods, the weighted average score‐level data fusion attains the highest classification accuracy (96.67%) for this dataset in the diagram (Figure 4f). The classification accuracy suggests that score‐level fusion may outperform feature‐level fusion for data types that greatly vary in dimensions. Although sleep posture recognition has been extensively explored either using a triboelectric mat array or infrared imaging sensor, a feasible strategy to further improve the classification accuracy has been effectively designed here. The multimodal sensory intelligence and data fusion analytics can assist the TMMSS in achieving privacy‐preserving sleep posture estimation under thick covers. This approach can be applied in comprehensive sleep monitoring and assessment described later in the text.

## 4 Application of TMMSS for Sleep Monitoring

The infrared imaging sensor and triboelectric mat array complement each other to create a multimodal sensory system capable of unobtrusive real‐time sleep posture and position recognition. Hence, a comprehensive sleep monitoring interface is demonstrated to show the applications of the integrated system under a smart home scenario. During its operation, a user sleeps on the triboelectric mat array while the infrared imaging sensor above captures thermal data. This system enables users to obtain a comprehensive record of bodily movements, the duration of each sleep stage, and detailed diagrams depicting the distribution of contact areas throughout the whole night. To perform comprehensive sleep monitoring, studying sleep cycles is pivotal for advancing knowledge in health sciences, improving clinical practices for sleep‐related disorders, and enhancing the overall health and productivity of individuals. The sleep cycle, consisting of awakeness, light sleep, and deep sleep, shows a progressively weaker intensity in human motor activity and a decrease in environmental awareness as the cycle continues.

**Figure 5:**
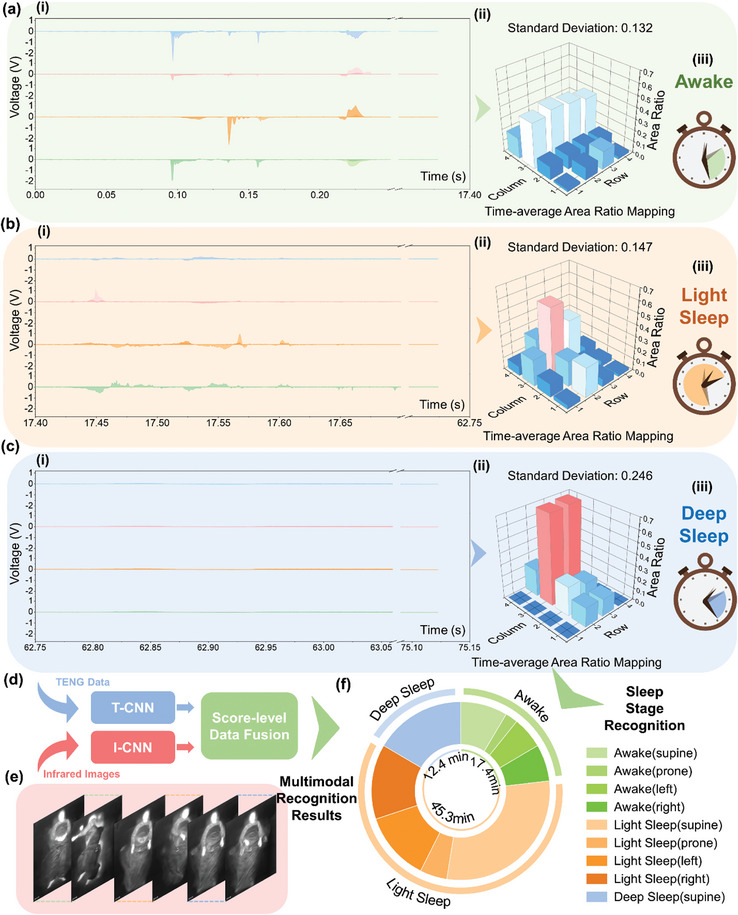
Demonstration of TMMSS in the application of a sleep monitoring interface. a) i) The triboelectric outputs generated by the mat array, ii) corresponding time‐average area‐ratio mappings (The detailed calculation principle of 3D diagram is depicted in Figure S14, Supporting Information) iii) from 0:10 AM to 0:27 AM. b) i) The triboelectric outputs generated by the mat array and ii) corresponding time‐average area‐ratio mapping iii) from 0:27 AM to 1:12 AM. c) i) The triboelectric outputs generated by the mat array and ii) corresponding time‐average area‐ratio mapping iii) from 1:12 AM to 1:25 AM. d) Simplified architecture for score‐level data fusion. e) Thermal images captured by the infrared imaging sensor from 0:10 AM to 1:25 AM (Figure S15–S17, Supporting Information). f) Comprehensive sleep monitoring diagram of the user's graphical interface. The time distribution for 4 sleep postures during different stages in one sleep cycle is detected in Figure S18 (Supporting Information). Finally, a more advanced sleep monitoring diagram can be generated containing the time distribution of all sleep stages in one cycle and 4 sleep postures (supine, prone, left, and right) in each sleep stage.

First, **Figure** **5**ai displays the triboelectric data for the initial 17 min when the user lies down. It is evident that during this period, from 0:10 AM to 0:27 AM, the voltage output exhibits significant absolute values and pronounced variations. This indicates that the user's body movements are relatively intense, with frequent changes in sleeping posture. By mapping and averaging the area ratios for each event, and representing these in a 3D plot, the time‐domain averaged area ratio mapping is derived, shown in Figure 5aii. The specific calculation process is detailed in Figure S14 (Supporting Information). Considering vigorous bodily behavior and extensive sleep‐covering area, the time‐average result should be more uniform for each row. In this regard, it can be inferred that the user is in a state of wakefulness based on the lowest standard deviation (0.13) among 3 sleep stages (Figure 5aiii). For the second sleep state, as shown in Figure 5bi, the voltage output amplitude decreases, and the variations slow down. This indicates that between 0:27 AM and 1:12 AM, the user's body activity diminishes, posture changes become less frequent, and the user gradually enters sleeping states, becoming less sensitive to external changes. Meanwhile, the corresponding time‐average mapping diagram in Figure 5bii becomes less uniform and more concentrated in the center region, which indicates the gradual bodily behaviors and concentrated sleep coverage area. According to a median absolute value of standard deviation (0.147), it can be included that the user is in the state of light sleep from 0:27 AM to 1:12 AM (Figure 5biii). Finally, Figure 5ci shows a near‐zero voltage output, with the corresponding area ratio mapping depicted in Figure 5cii. This figure demonstrates a very localized area distribution, indicating that there are no significant changes in motion and the body position remains unchanged. Hence, based on the highest value of standard deviation (0.246), the third sleep stage can be classified as deep sleep (Figure 5ciii). Ultimately, a method that analyzes only the triboelectric data is employed to generate the time‐domain averaged area ratio mapping and calculate its standard deviation to achieve sleep stage recognition.

Simultaneously, the TMMSS utilizes an infrared imaging sensor positioned above the triboelectric mat array to provide thermal images, which are combined with triboelectric signals to enable multimodal recognition. Figure 5e presents corresponding thermal data within one sleep cycle, with detailed data in Figures S15–S17 (Supporting Information). To monitor different sleeping postures across various sleep stages, a multimodal DL network is employed like that shown in Figure 4e(ii), as illustrated in Figure 5d. In this network, thermal images and TENG data serve as the input for I‐CNN and T‐CNN respectively. Furthermore, two generated possibility vectors are sent into a score‐level data fusion network to get 4 sleep posture multimodal recognition results. The Recognition time distribution diagrams for the 3 stages are shown in Figure S18a–c (Supporting Information). Finally, a more sophisticated sleep monitoring chart can be produced that includes the time distribution of all sleep stages within one cycle, as well as the four sleep postures (supine, prone, left, and right) within each sleep stage in Figure 5f. This comprehensive sleep monitoring interface, designed for smart home applications, gives users a detailed analysis report about the time distribution of different sleep postures in each stage while ensuring fully protected privacy.

## 5 Digital‐Twin Applications of TMMSS in Smart Home

Apart from the comprehensive sleep monitoring interface, the TMMSS can also realize digital‐twin smart homes via interactions between the real world and virtual space. In this regard, daily activities can be recognized and projected into virtual space, including emotion‐relevant and unemotional ones. These functionalities are based on integrating thermal images captured by an infrared imaging sensor and triboelectric information generated by the mat array in the TMMSS.

**Figure 6:**
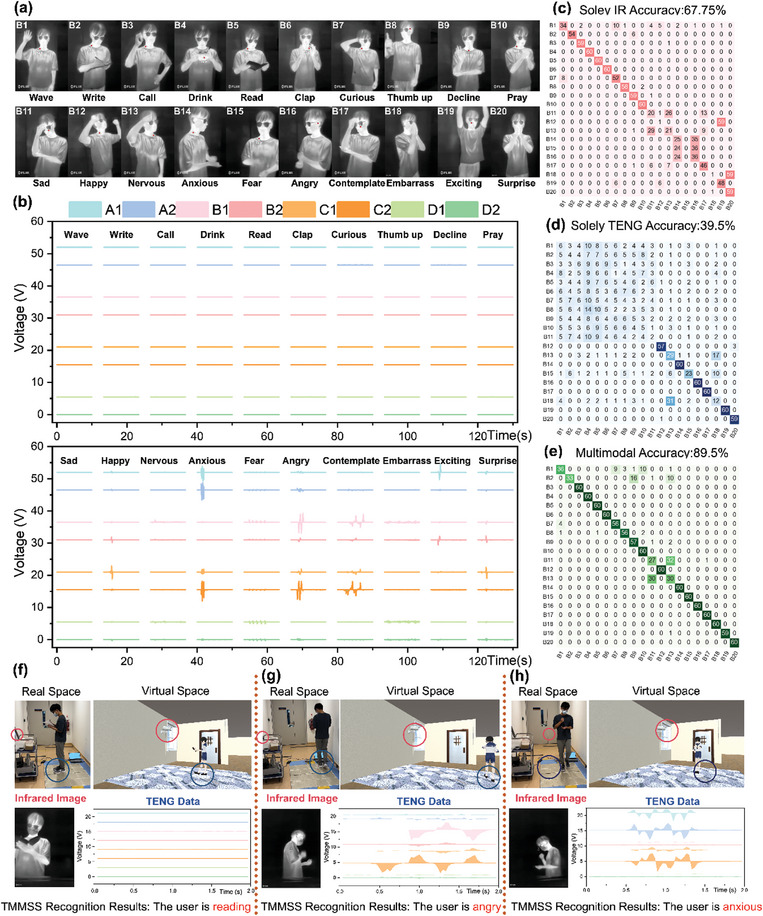
The TMMSS for digital‐twin smart home applications. a) Thermal images of 20 behaviors captured by the infrared imaging sensor in a fixed location. These images display the temperature distribution and capture the movements of the upper body. b) The triboelectric voltage of 20 behaviors produced by the triboelectric mat array. These results involve users' gait information. c) Behavior recognition results for thermal data only. The corresponding t‐SNE diagram is shown in Figure S19a (Supporting Information). d) Behavior recognition results for triboelectric information only. The t‐SNE diagram is shown in Figure S19b (Supporting Information). e) Behavior recognition results of multimodal data fusion analytics (detailed parameters of the multimodal CNN networks in Figure S20, Supporting Information). The t‐SNE diagram is shown in Figure S19c (Supporting Information). f–h)Demonstration of different behaviors (“read”, “angry”, and “anxious”), where the user is in the TMMSS, while his digital twin is controlled in virtual space accordingly. Therefore, the TMMSS can accurately distinguish these emotional‐relevant behaviors (“angry” and “anxious”) and enable a digital‐twin projection in virtual space.

First, 20 common behaviors have been selected from daily life, including those related to user emotions, such as happiness, nervousness, anger, etc., and behaviors not related to user emotions, such as reading, drinking, etc. Then a fixed infrared imaging sensor is utilized to capture the thermal data of a user performing these 20 behaviors, as shown in **Figure** **6**a. The camera primarily captured the user's upper body motions. In these images, the user's skin appears bright due to higher temperature, while the environment appears black due to lower temperature, effectively preserving the privacy of the home environment. From this data, some actions have distinct characteristics, such as “Wave,” “Write,” “Call,” “Drink,” “Read,” “Clap,” “Curious,” “Thumb up,” “Decline,” and “Pray.” Some upper limb behaviors related to emotions exhibit similarities. For instance, both “Happy” and “Exciting” involve relatively relaxed upper limb movements, including raised arms and upward‐tilted head positions. Similarly, “Angry” and “Anxious” thermal data also share similarities, such as tightly clenched fists and tense muscles. Meanwhile, users in a “Surprise” state or “Embarrass” state tend to cover their mouths with their hands. Users in an “Angry” or “Contemplate” state often lower their heads and support them with their hands. The thermal data forms the dataset, and the corresponding t‐SNE result is shown in Figure S19a (Supporting Information). The classification results using a CNN network are shown in Figure 6c. Due to the similarity in upper limb behaviors associated with emotion‐relevant activities, the classification results are not ideal, with only 67.75% accuracy if only based on thermal information. Therefore, triboelectric data is also needed for sensing to complement the missing lower limb information.

Meanwhile, triboelectric information has been collected for these 20 behavior characteristics, as shown in Figure 6b. It is evident that the first 11 states do not involve lower limb movements, so no corresponding data was collected. Likewise, some gait information collected from users exhibits similarities. When users are in “happy” or “surprise” states, the corresponding gait information shows a positive voltage followed by a negative voltage, indicating that users leave and then contact the same mat, suggesting a jumping motion. When users are in “nervous,” “fear,” or “embarrass” states, the triboelectric output shows many small amplitude peaks, indicating that the corresponding gait is relatively chaotic and involves shuffling. When users are “anxious” or “angry,” the triboelectric data shows larger absolute values, indicating more intense lower limb movements. The corresponding t‐SNE data is shown in Figure S19b (Supporting Information). The classification results using a CNN network are shown in Figure 6d. Due to the lack of upper limb behavior information, the classification results are also not ideal, with only 39.5% accuracy. Therefore, the upper limb information should be provided by the infrared imaging sensor as a supplement.

Considering limited upper body motions in triboelectric outputs and insufficient gait information in thermal images, integrating thermal images and triboelectric data might be a promising solution for supplementary. To validate the viability of the TMMSS, the perfect visualization diagram in Figure S19c (Supporting Information) shows strong categorization capability with almost all class points being distinctly distributed. When combining the TMMSS with DL methods and data fusion analytics, a digital‐twin smart home can be realized for daily behavior, especially emotion‐relevant activity recognition and projection into the virtual world. As is shown in Figure 6e, multimodal CNN (detailed information in Figure S20, Supporting Information) gets the highest test accuracy of 89.5% for 20 labels, thereby it is a feasible strategy in digital‐twin smart home applications.

Unlike the digital camera monitoring normally involving video‐taken concerns, this TMMSS uses a digital twin of the person in the virtual environment showing the physical and emotional information, which is the fundamental element for smart home applications. When a user stands on the mat array with no electrical outputs generated, an infrared imaging sensor on the wall captures images simultaneously. The multimodal data can be adopted as the trigger signal to control the digital twin, and the full cycle multimodal signal is then analyzed using the DL fusion analytics model to predict the behavior of user, as shown in Figure 6f. Scenarios with similar sensory signals are illustrated in Figure 6g,h to illustrate the robustness of the multimodal system. When a user is stepping heavily and gripping the fist firmly, corresponding multimodal data is shown in Figure 6g. Meanwhile, when a user is in an irregular and uneasy pace with similar upper body motions, corresponding signals are illustrated in Figure 6h. Finally, the TMMSS can perfectly distinguish these emotional‐relevant behaviors (“angry” and “anxious”) and enable a digital‐twin projection in virtual space.

## 6 Conclusion

The metaverse plays a significant role in mapping real‐world objects into virtual spaces through AR and VR technologies. In this regard, digital‐twin smart homes have emerged as a crucial domain, a vital bridge between the virtual world and reality. To achieve comprehensive home monitoring, protecting user privacy has become a critical consideration for sensors in smart home applications. To address this problem, a key component within this domain is the triboelectric mat, an important self‐powered distributed sensor that can unobtrusively convert users' gait information into electrical signals. When combined with DL algorithms, it enables functions such as identity recognition and position tracking. Unlike traditional floor mats that overlook the capture of upper body information, the TMMSS consisting of a commercial infrared imaging sensor and a triboelectric floor mat array sensor is designed and fabricated. This system can simultaneously capture upper limb movements and gait information. By integrating advanced multimodal DL algorithms, it achieves comprehensive sleep monitoring, emotion‐relevant activity monitoring, and mapping to the virtual world.

Specifically, each mat pixel in our system is designed with universal IDEs, whose area ratio is 8:2. By employing different wiring methods, 4 types of voltage ratio outputs can be achieved (0:10, 2:8, 8:2, and 10:0). Using preset voltage ratio thresholds, different voltage ratio mats can be identified by distinguishing the negative peak ratios accurately. Our 4 × 4 triboelectric mat array features a minimalistic design, utilizing interval connection methods to optimize system complexity without compromising the accuracy of daily motion recognition. Benefiting from voltage integration calculations, real‐time sleep position area ratio mapping can be achieved solely through the triboelectric mat array without concerns about personal privacy. Subsequently, thermal sleep data combined with the TENG mat array data is introduced for multimodal analysis. In the multimodal CNN, the weighted average score‐level data fusion technique is introduced, achieving the highest classification accuracy (96.67%) across 12 labels involving 4 sleeping postures for 3 users. This system can provide a comprehensive sleep monitoring interface based on the integration of area ratio mapping and multimodal sleep posture classification. By analyzing the area ratio mapping of triboelectric data during the sleep cycle of a user and calculating the corresponding standard deviation, TMMSS can determine which sleep stage (Awake, Light Sleep, and Deep Sleep) the user is in. Combining this with multimodal sleep posture classification using triboelectric and thermal data for each sleep stage enables comprehensive sleep health monitoring. Meanwhile, unlike most sensing systems that can only detect physical behaviors, our TMMSS can recognize and map emotion‐relevant behaviors into the virtual space. Through the analysis of multimodal data, the system can classify 20 types of daily behaviors (including both emotion‐related and non‐emotion‐related behaviors) using multimodal DL algorithms, achieving an accuracy of 87%, significantly surpassing the accuracy of using only the triboelectric array or only the infrared imaging sensor. Therefore, this cost‐effective, non‐intrusive system holds great potential for various digital‐twin smart home applications in the metaverse, such as healthcare and sports.

## 7 Experimental Section

### Fabrication of the Triboelectric Mat Pixel

The triboelectric mat consists of four layers: a polyethylene terephthalate (PET) top triboelectric layer (125 µm thick), a silver (Ag) electrode layer, a polyvinyl chloride (PVC) layer, and a thermoplastic elastomer (TPE) supporting layer. The process begins with cutting the PET film into rectangular pieces with the size of 340 mm × 290 mm. These pieces are treated with a primer to enhance adhesion with subsequent layers. Next, silver paste, chosen for its excellent conductivity and printability, is screen‐printed on the PET using a universal and predesigned mask, allowing uniform application across all mat pixels. The printed electrode area measures 324 mm × 270 mm and includes two output electrodes. Following printing, the electrode layer undergoes thermal curing at 130 °C for 30 min in an oven, resulting in a silver layer thickness of 15 µm. The PET‐Ag film is then cold‐laminated with an 80 µm thick PVC film, positioning the Ag paste electrode between the PET and PVC layers. This composite structure is subsequently bonded to a 1.5 mm TPE layer using double‐sided adhesive Kapton tape. Copper wires are connected to the electrodes by peeling off a small section of the PVC layer to expose them. Upon completion, 4 mat configurations are produced (0:10, 2:8, 8:2, and 10:0) for constructing a mat array. The following section discusses the data collected from the fabricated mats in various activities and the corresponding recognition performance.

### Characterization of the Experimental Outputs

The output voltage of the triboelectric mat array is measured and recorded by an oscilloscope (Agilent DSO‐X3034A) with the impedance of 1MΩ. The thermal data is collected using the FLIR ONE PRO, a commercial infrared imaging sensor, positioned at a constant height.

### Triboelectric Data Collection and Deep‐Learning Analytics

The triboelectric voltage signals produced by the triboelectric mat array are continuously collected and processed in real‐time by the Arduino MEGA 2560 microcontroller. The convolutional neural network (CNN) model is developed using Python, leveraging Keras with a TensorFlow backend.

### Sleep Posture Recognition based on the TMMSS

3 users' 4 sleep postures, a total of 12 labels are applied for performing sleep posture recognition, including “supine1”, “prone1”, “left1”, “right1”, “supine2”, “prone2”, “left2”, “right2”, “supine3”, “prone3”, “left3”, and “right3”. In the measurement, the user is lying on the triboelectric mat array with the infrared imaging sensor positioned above. The resulting triboelectric and thermal datasets contain 50 data samples for each activity, while 40 and 10 samples are randomly picked for training and testing.

### Emotion‐Relevant and Non‐Emotional Activity Recognition and Digital Twin Smart Home Applications based on TMMSS

20 different kinds of daily activities containing emotion‐relevant and non‐emotional ones are applied for performing the activity classification, including “Wave”, “Write”, “Call”, “Drink”, “Read”, “Clap”, “Curious”, “Thumb up”, “Decline”, “Pray”, “Sad”, “Happy”, “Nervous”, “Anxious”, “Fear”, “Angry”, “Contemplate”, “Embarrass”, “Exciting”, and “Surprise”. 10 of them (“Sad”, “Happy”, “Nervous”, “Anxious”, “Fear”, “Angry”, “Contemplate”, “Embarrass”, “Exciting”, and “Surprise”) are directly related to the emotions. In the measurement, the user is walking on the triboelectric mat array with the infrared imaging sensor positioned above. The resulting triboelectric and thermal datasets contain 300 data samples for each activity, while 240 and 60 samples are randomly picked for training and testing. Then the multimodal processed signals are sent to the VR scenario which is developed based on 3D Unity for control.

## Conflict of Interest

The authors declare no conflict of interest.

## Author Contributions

J.L.X. and X.G.G. contributed equally to this work. J.L.X. and C.K.L. conceived the idea. J.L.X. and Z.X.Z. carried out the device fabrication. J.L.X. conducted the device characterization and performed the experiments. J.L.X. and X.G.G. contributed to the data analysis and figures plot. J.L.X. drafted the manuscript. X.G.G., Z.X.Z., H.J.L., and C.K.L. edited the manuscript.

## Supporting information



Supporting Information

## Data Availability

The data that support the findings of this study are available from the corresponding author upon reasonable request.
