# Supplementary material for: Triboelectric Mat Multimodal Sensing System (TMMSS) Enhanced by Infrared Image Perception for Sleep and Emotion‐Relevant Activity Monitoring
Source: Adv Sci (Weinh). 2024 Dec 19;12(6):2407888. doi: 10.1002/advs.202407888 (PMC11809373; doi:10.1002/advs.202407888)
Supplement: Supplementary file 1 — Supporting Information [file ADVS-12-2407888-s001.docx]

Supplementary Materials

**Triboelectric Mat Multimodal Sensing System (TMMSS) Enhanced by Infrared Image Perception for Sleep and Emotion-Relevant Activity Monitoring**

*Jinlong Xu^*^, Xinge Guo^*^, Zixuan Zhang, Huajun Liu^#^, and Chengkuo Lee^#^*

The file includes:

**Figure S1.** The voltage ratios for (a) different weight and (b) different humidity.

**Figure S2.** The block diagram illustrates the operational procedures and data flow to estimate sleep posture.

**Figure S3.** Precise dimension of the electrode pattern.

**Figure S4.** Investigation of electrical outputs with varying voltage ratio pixels.

**Note S1.** The principle for calculating the preset voltage ratio threshold varies for different pixel classifications.

**Figure S5.** The detailed electrode connection for different channels.

**Figure S6.** The digital image of the multifunctional mat array with 4*4 pixels.

**Figure S7.** The CNN model structure for different motion classifications.

**Figure S8**. The confusion maps of different channel numbers.

**Figure S9.** The electrical outputs and corresponding integral values under different speeds.

**Note S2.** The calculating principle of average voltage integral values when contacting the whole area of one pixel**.**

**Figure S10.** The illustration of CNN structure for infrared image classification.

**Figure S11.** The illustration of CNN structure for triboelectric data classification.

**Figure S12.** The illustration of CNN structure for multimodal perceptions.

**Figure S13.** The confusion maps for feature-level and weighted score-level data fusion.

**Figure S14.** The illustration of three-dimensional time-average area ratio mapping calculation procedures.

**Figure S15.** Infrared images are measured when the user is awake.

**Figure S16.** Infrared images are measured when the user is in the light sleep state.

**Figure S17.** Infrared images are measured when the user is in the deep sleep state.

**Figure S18.** The time distribution for 4 sleep postures during different stages in one cycle.

**Figure S19.** The t-SNE figures for different outputs.

**Figure S20.** The detailed parameters of the neural network for multimodal CNN.

**Table S1.** Comparison of smart mats or mattresses for smart home applications.

**Note S3.** Calculating the area ratio of each pixel by matrix computations.

**Table S2.** Summary of characters and corresponding descriptions used in the manuscript.

**Table S3.** Summary of abbreviations and full term used in the manuscript.


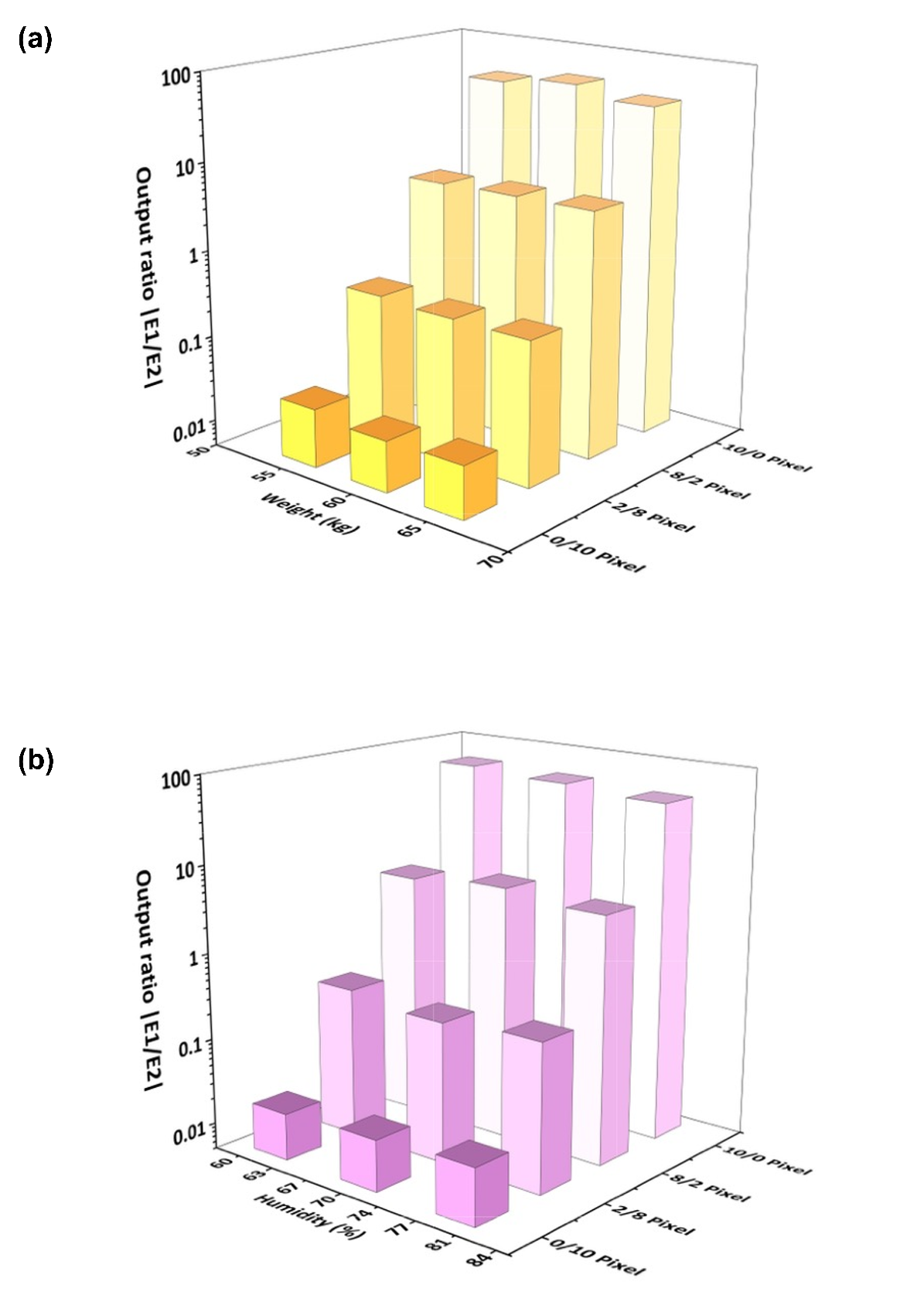


**Figure S1.** The voltage ratios for (a) different weight and (b) different humidity. The voltage ratios remain stable and identifiable across different pixels when the user's weight changes. Given that rising humidity increases air moisture, affecting TENG charging, the voltage ratios can counteract this impact, enhancing the distinctiveness of different ratio-based pixels.


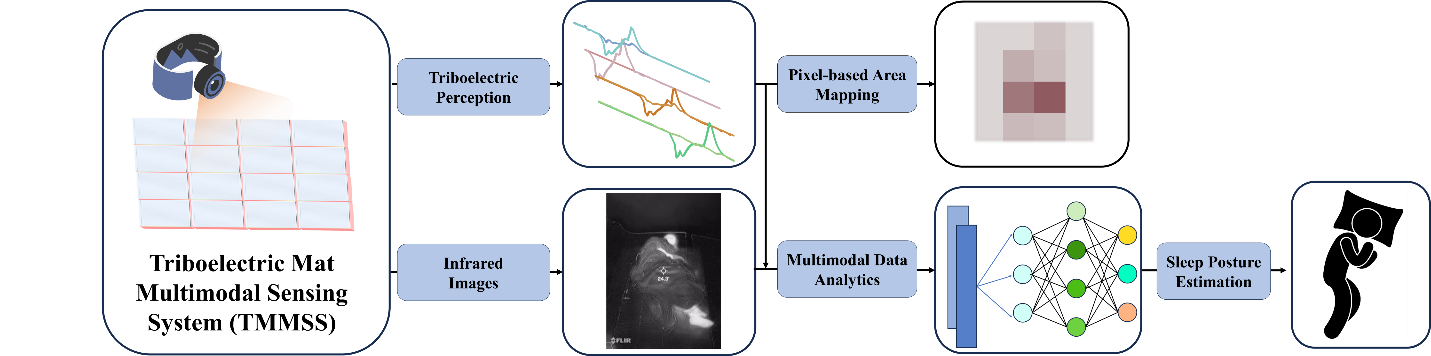


**Figure S2.** The block diagram illustrates the operational procedures and data flow to estimate sleep posture.


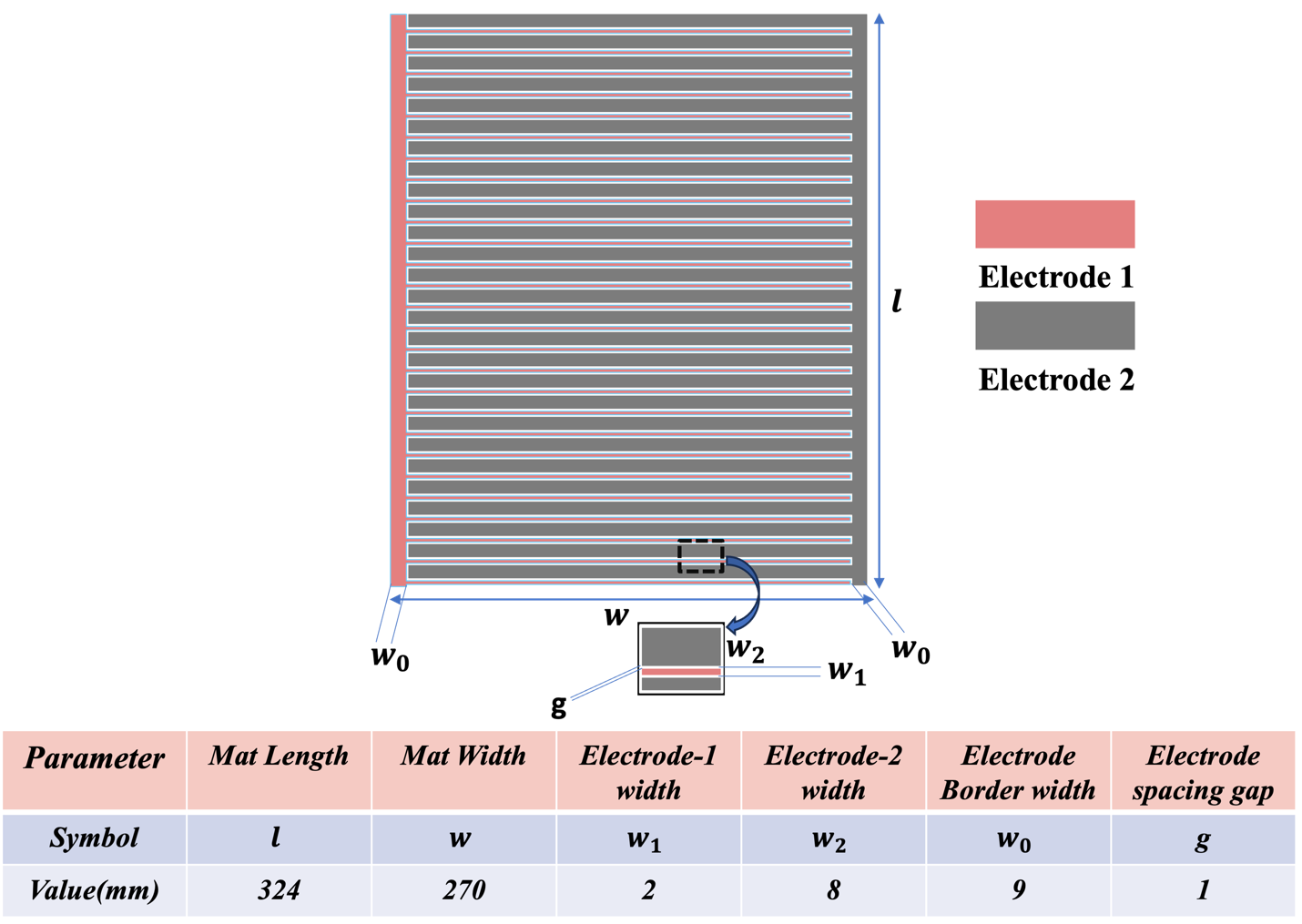


**Figure S3.** Precise dimension of the electrode pattern.


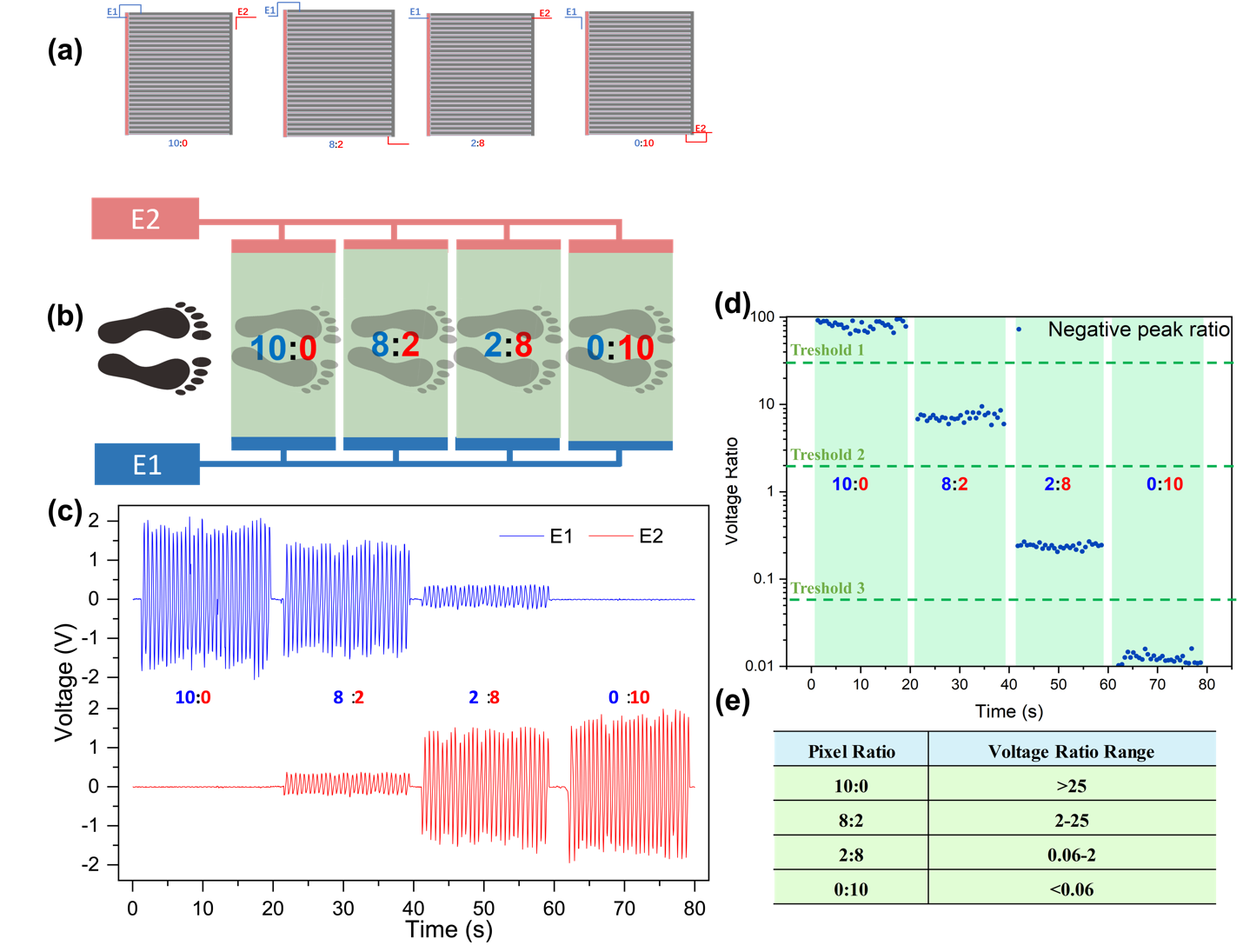


**Figure S4.** Investigation of electrical outputs with varying voltage ratio pixels. (a) The wiring connections for the 4 kinds of voltage ratio configurations. (b) The schematic diagram of the parallel connection between two electrodes. (c) The generated output voltages and (d) the corresponding negative peak-to-peak voltage ratio of 4 kinds of voltage ratio configurations. (e) The defined threshold ratio for the 4 pixels.

# **Note S1.** The principle for calculating the preset voltage ratio threshold varies for different pixel classifications.

For triboelectric contact-separation mode, the output voltage of TENG is directly proportional to the contact area, resembling the IDEs ratio under constant conditions. **Figure S3a** demonstrates that employing varied wiring strategies enables the creation of mats electrodes, capable of exhibiting a range of voltage ratios, including specific configurations like 0:10, 2:8, 8:2, and 10:0. **Figure S3b** illustrates the schematic diagram of the parallel connection of 4 mats with different voltage ratios. The generated output voltages and corresponding negative peak-to-peak voltage ratios under consistent stepping on and off conditions with shoes equipped with polytetrafluoroethylene (PTFE) soles are presented in **Figure S3c, d**. The consistent outputs exhibited by electrodes demonstrate repeatability, a key factor in ensuring uniform dependable performance in TENGs. The output ratios, as derived, exhibit a distinctly different distribution, allowing for precise separation with a proper threshold in **Figure S3e**.


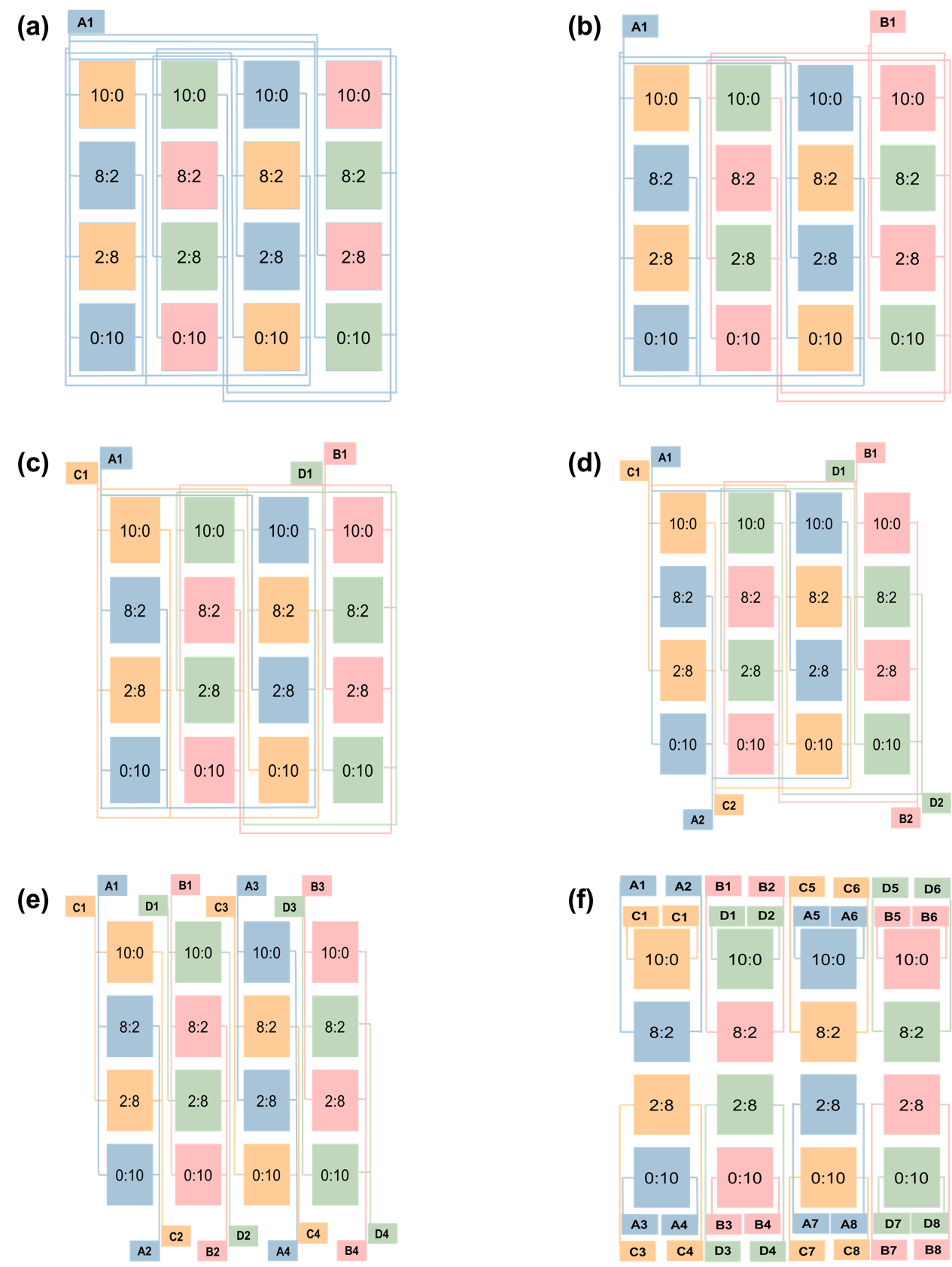
.

**Figure S5.** The detailed electrode connection for different channels. a) 1 channel, b) 2 channels, c) 4 channels, d) 8 channels, e) 16 channels, and f) 32 channels.


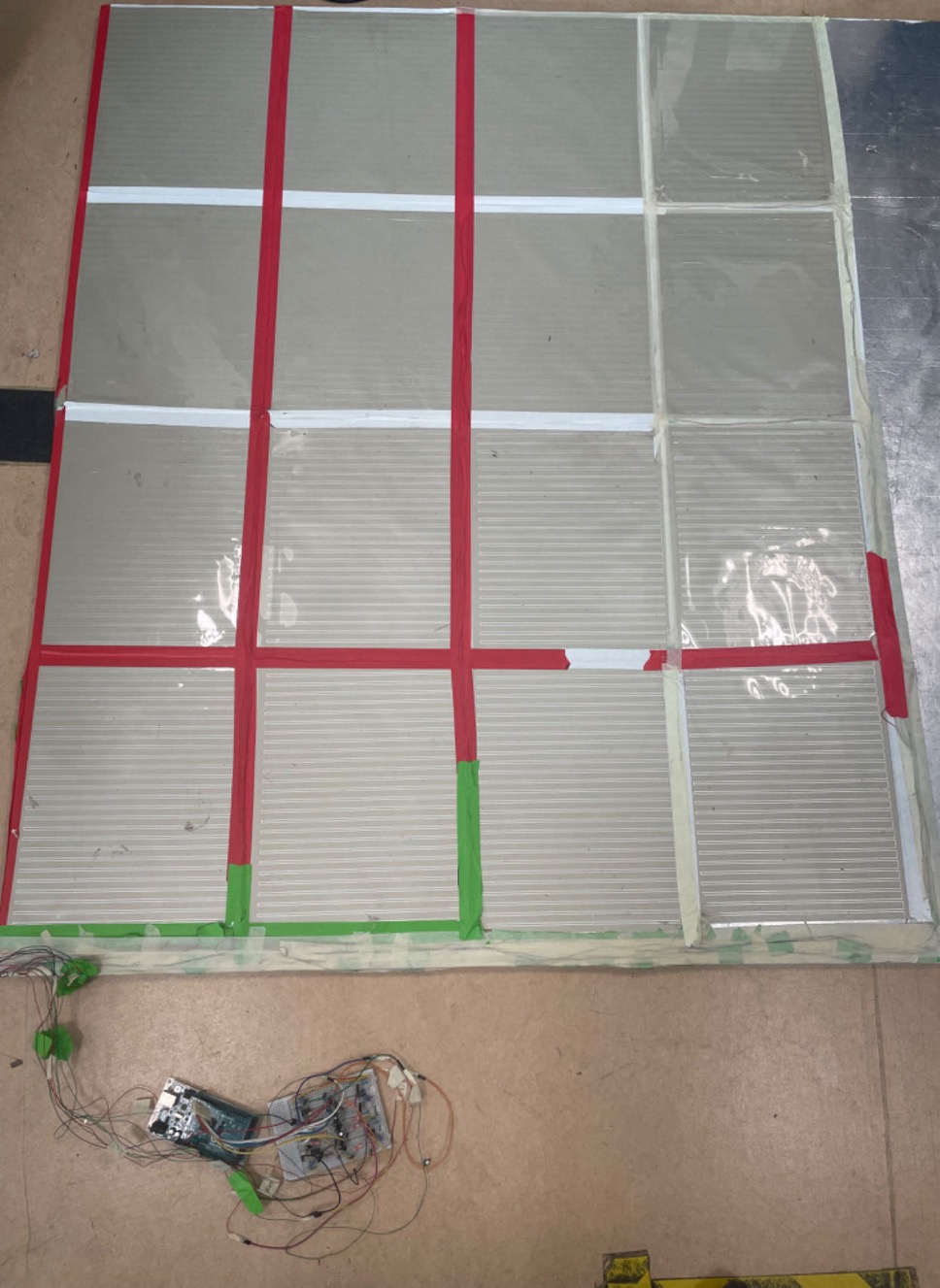


**Figure S6.** The digital image of the multifunctional mat array with 4*4 pixels


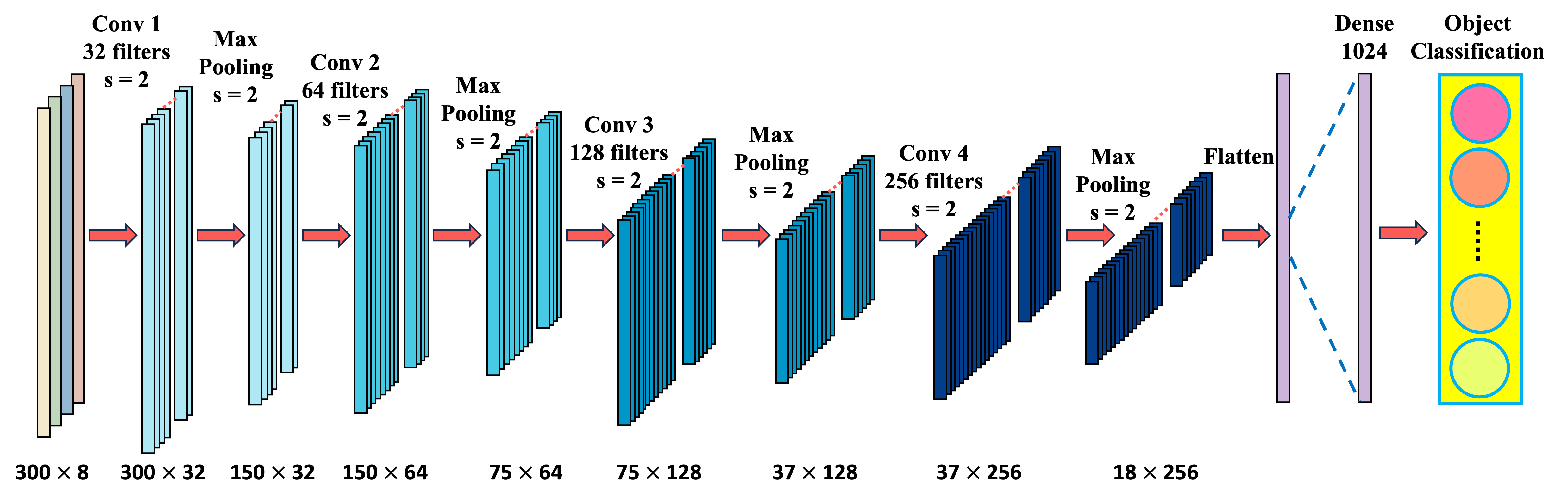


**Figure S7.** The CNN model structure for different motion classifications.


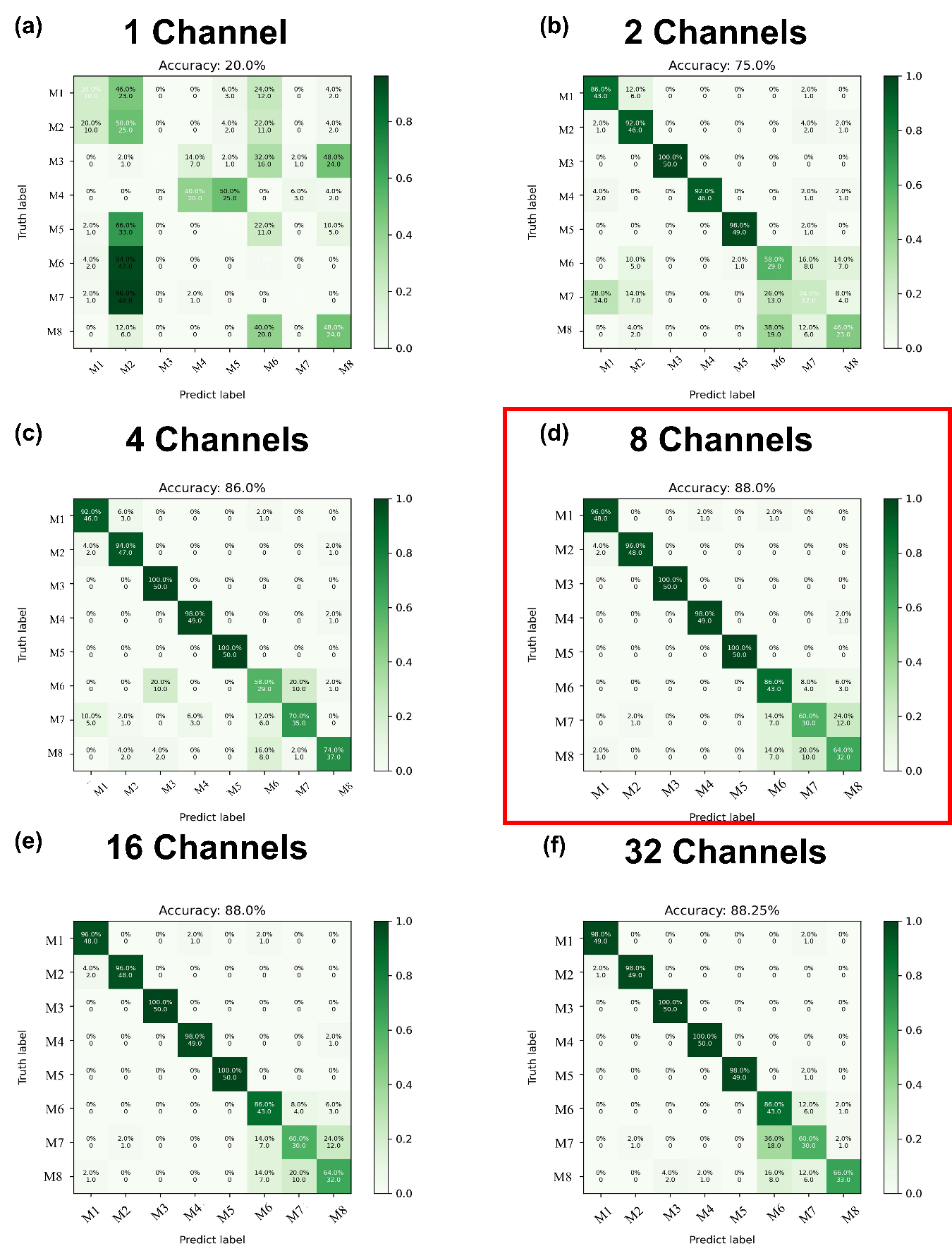


**Figure S8**. The confusion maps of different channel numbers. a) 1 channel, b) 2 channels, c) 4 channels, d) 8 channels, e) 16 channels, and f) 32 channels.


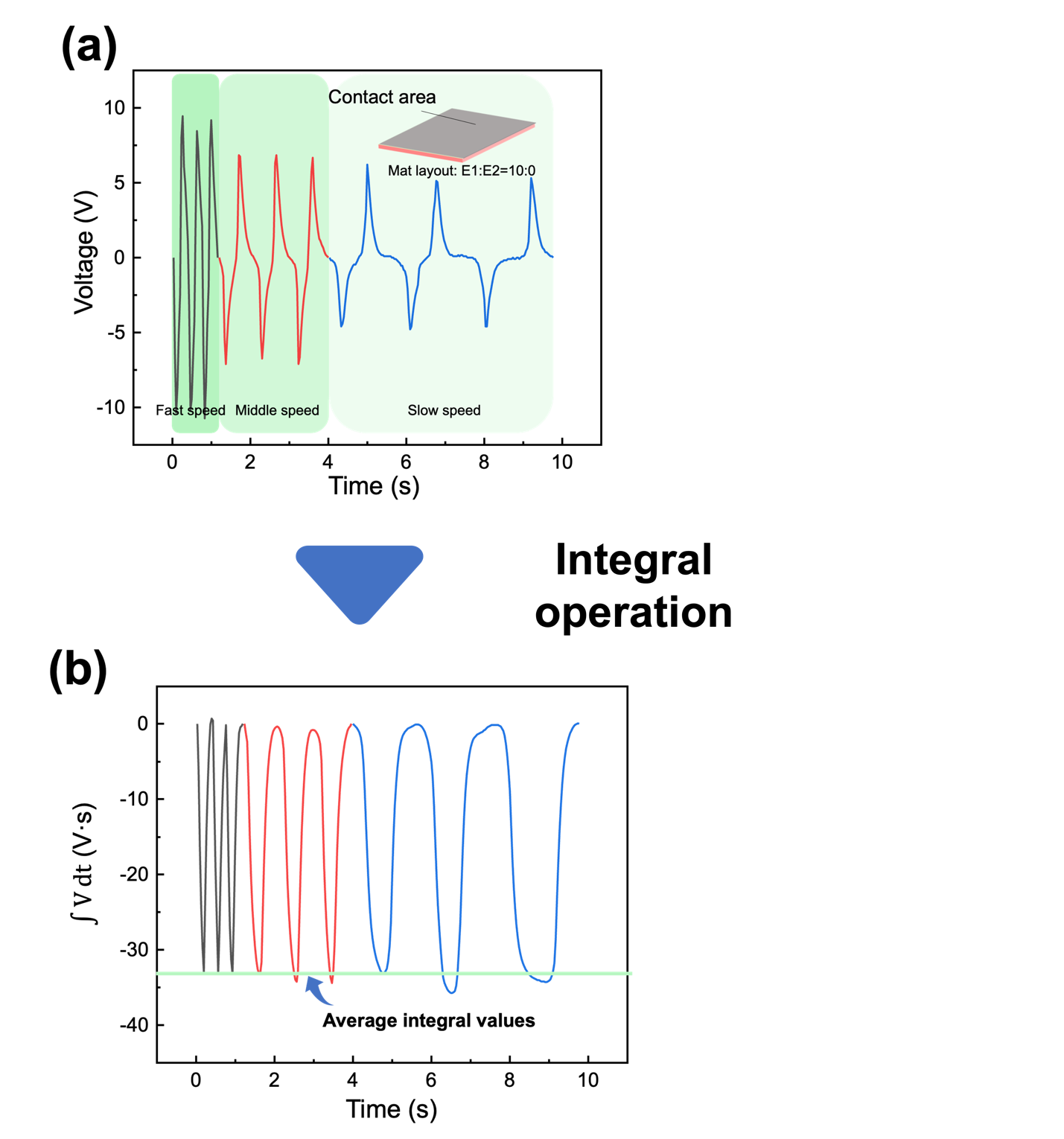


**Figure S9.** The electrical outputs and corresponding integral values under different speeds. a) The electrical outputs under different speeds and b) corresponding integral values.

# **Note S2.** The calculating principle of average voltage integral values when contacting the whole area of one pixel**.**

To investigate whether different contact speeds influence the voltage integral values, **Figure S8a** demonstrates the TENG outputs under constant contact of one mat pixel (E1:E2 = 10:0) with different speeds (fast, middle, and slow). Employing voltage integral operation, the integration values in **Figure S8b** defined as, which is proportional to the transferred charges in TENG contact-separation mode, are almost the same at negative peaks. Then we calculate the average minimum integral values Γ_min_ = -33 V·s, indicating the transferred charges while contacting the whole pixel area Λ_max_. In this regard, since transferred charges are proportional to the contact area Λ, we can use the ratio of voltage integration Γ/ Γ_min_ to quantify the contact area ratio Λ/ Λ_max_ for each pixel in sleep monitoring.


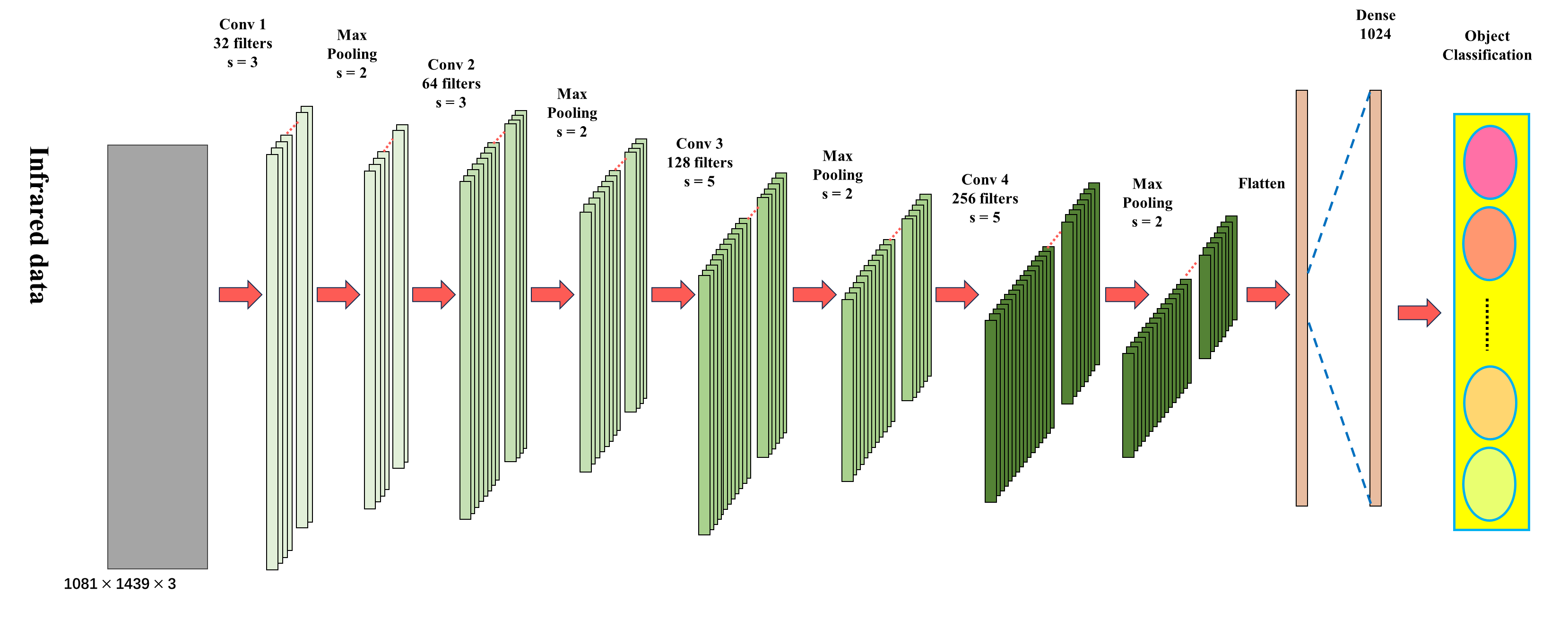


**Figure S10.** The illustration of CNN structure for infrared image classification.


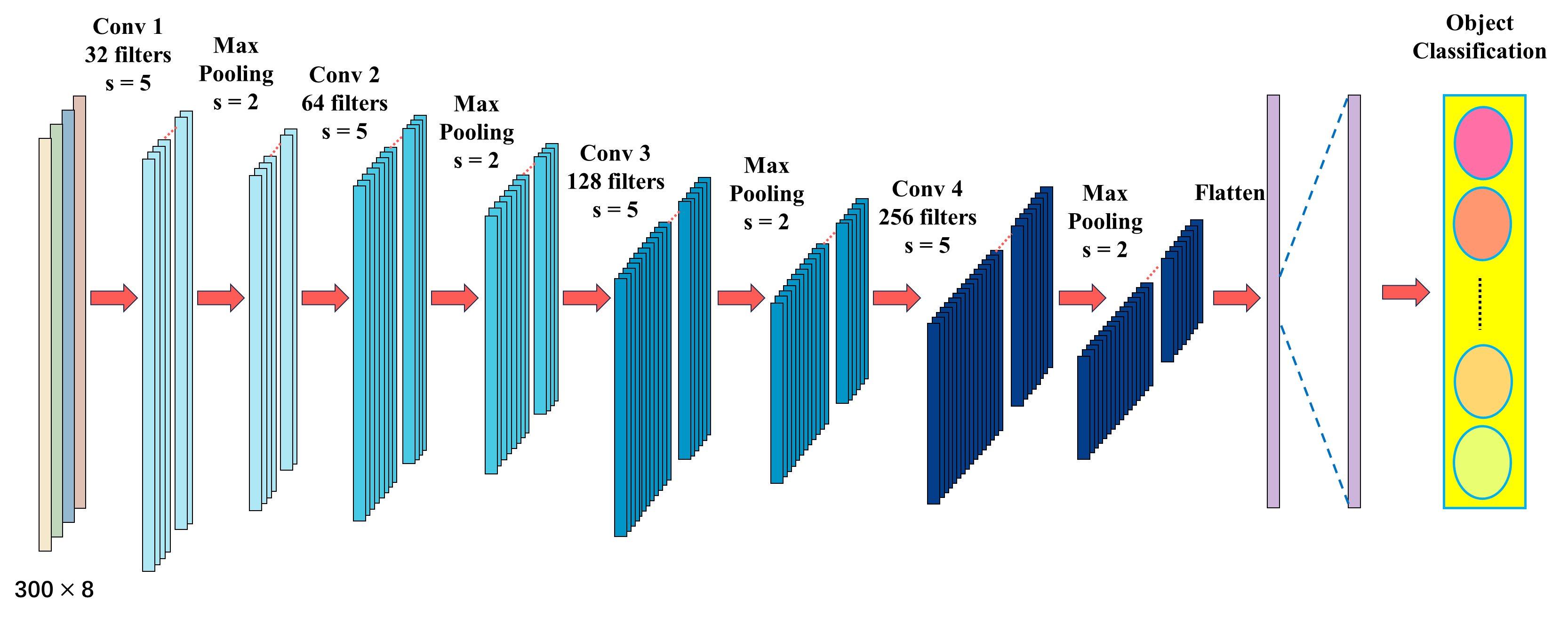


**Figure S11.** The illustration of CNN structure for triboelectric data classification.


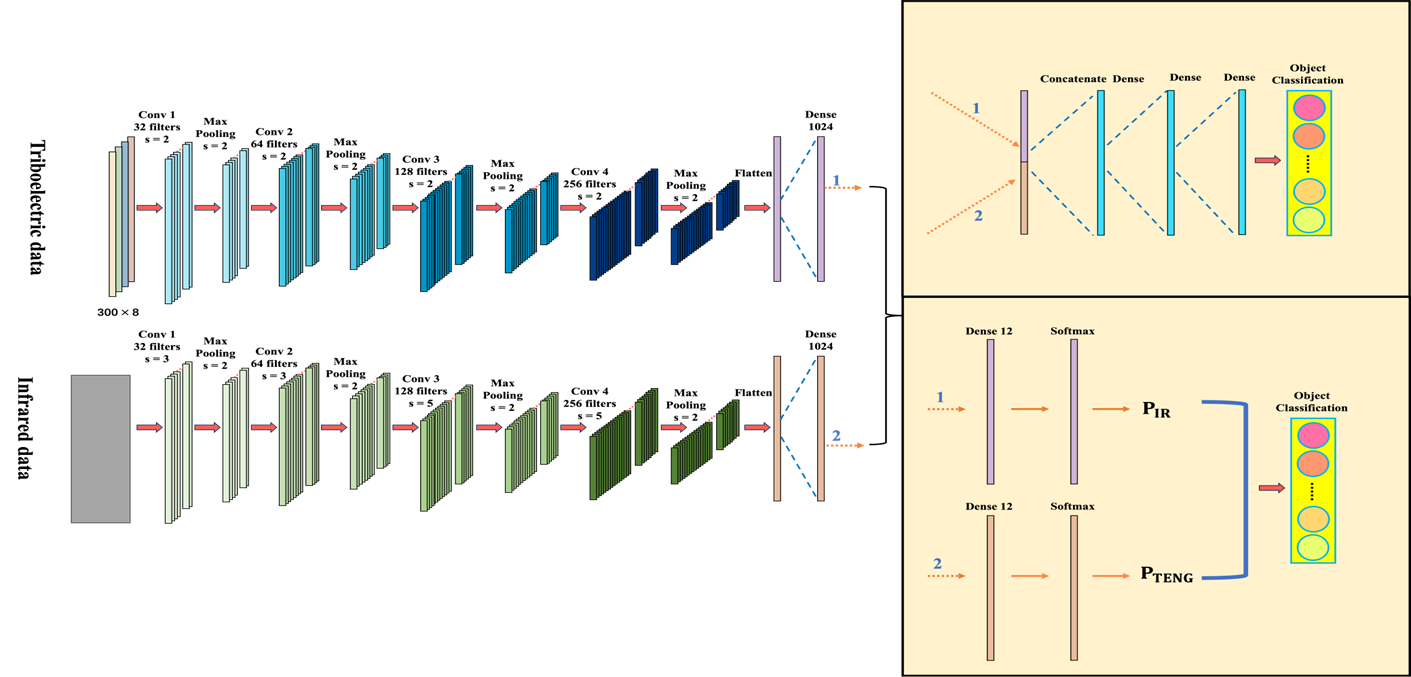


**Figure S12.** The illustration of CNN structure for multimodal perceptions. a) Feature-level data fusion b) Score-level data fusion.


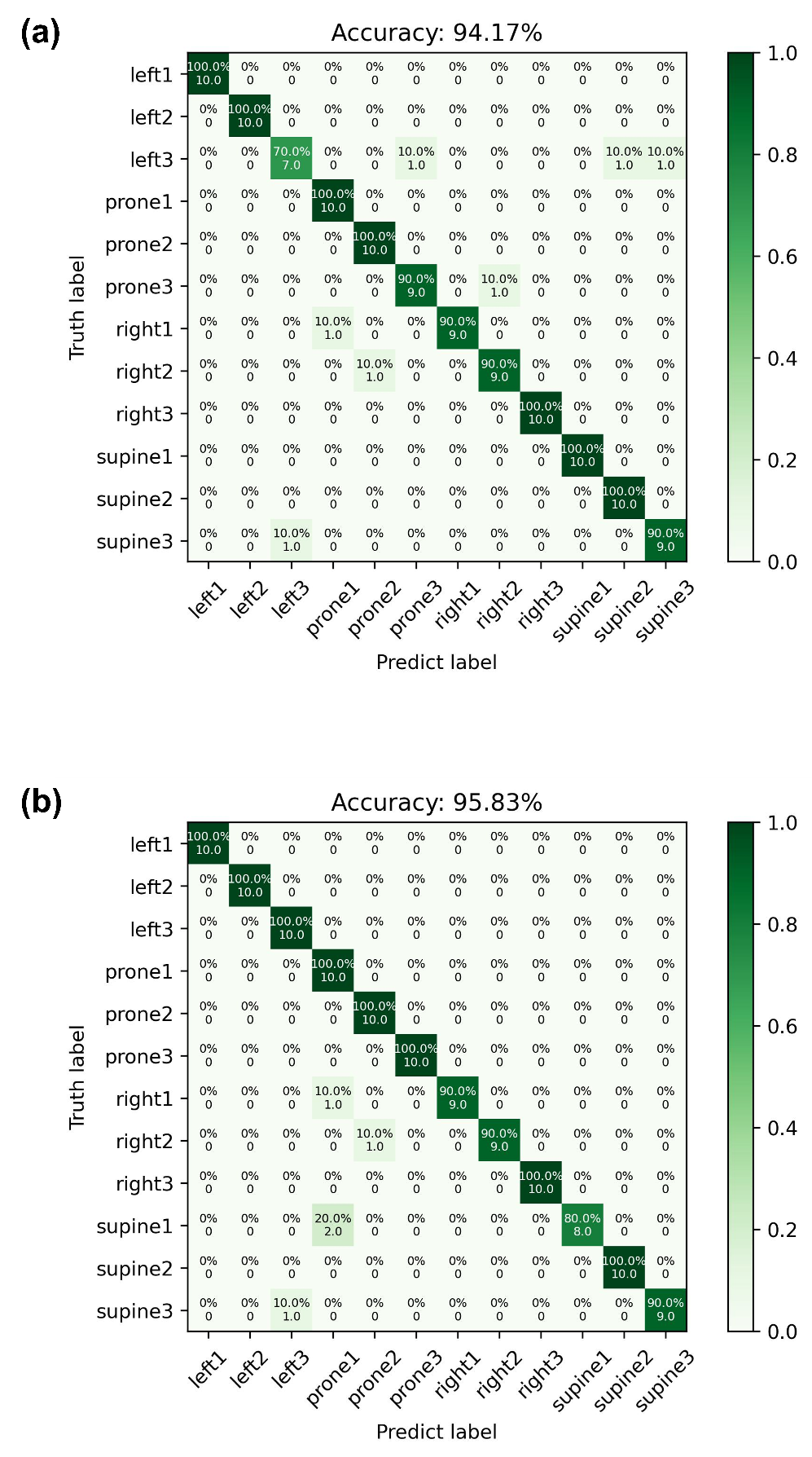


**Figure S13.** The confusion maps for feature-level and weighted score-level data fusion. The confusion map for a) feature-level data analysis and b) weighted data confusion method.


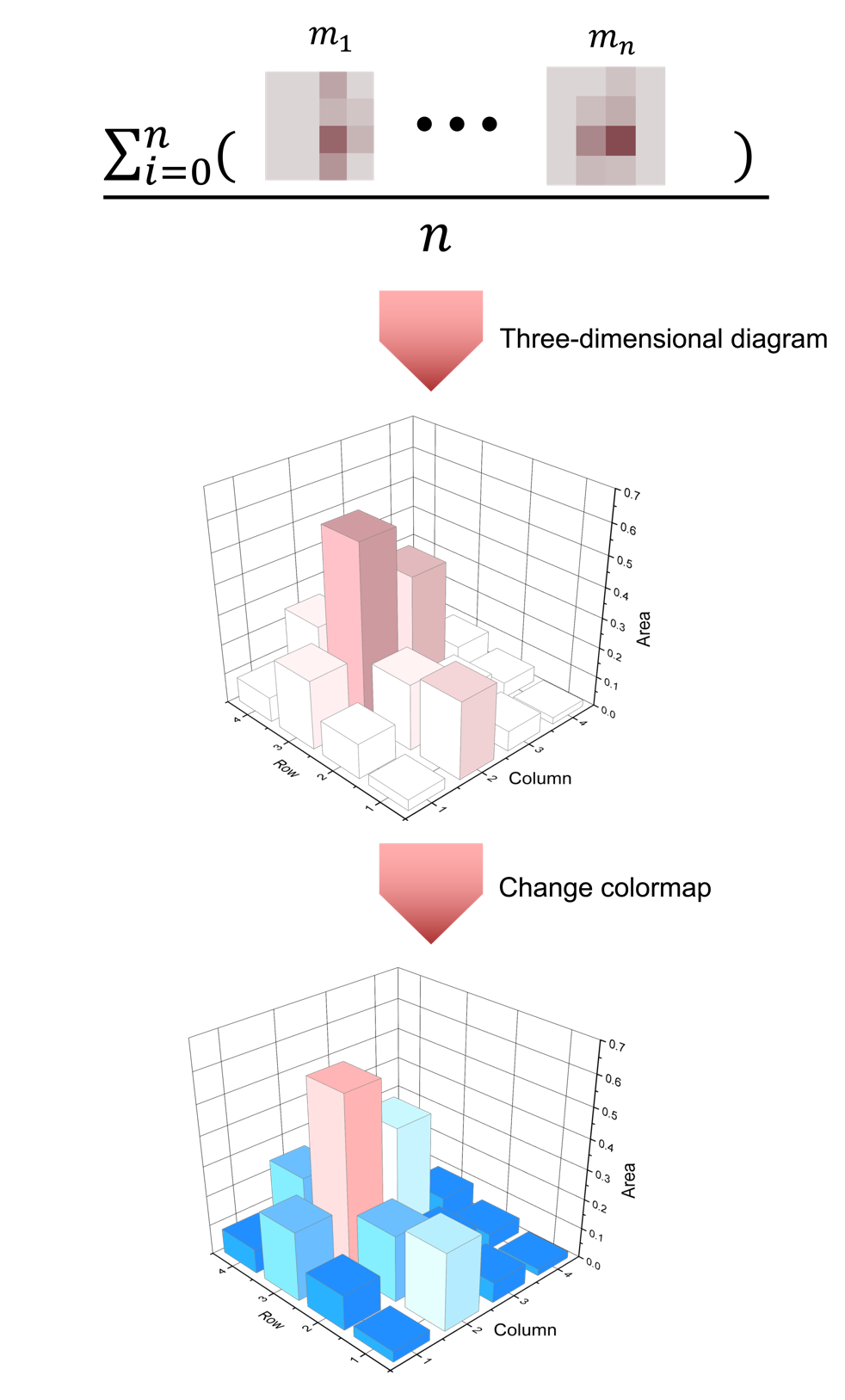


**Figure S14.** The illustration of three-dimensional time-average area ratio mapping calculation procedures.


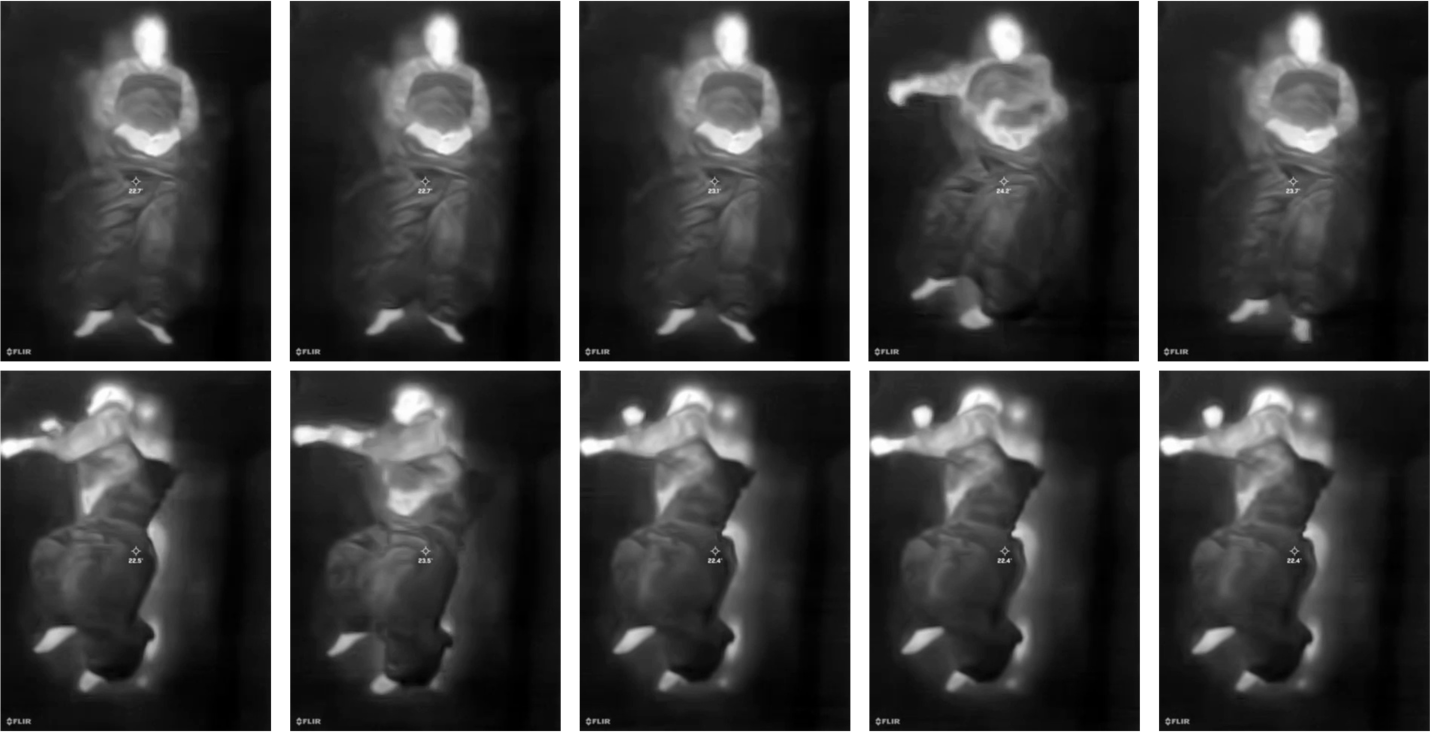


**Figure S15.** Infrared images are measured when the user is awake.


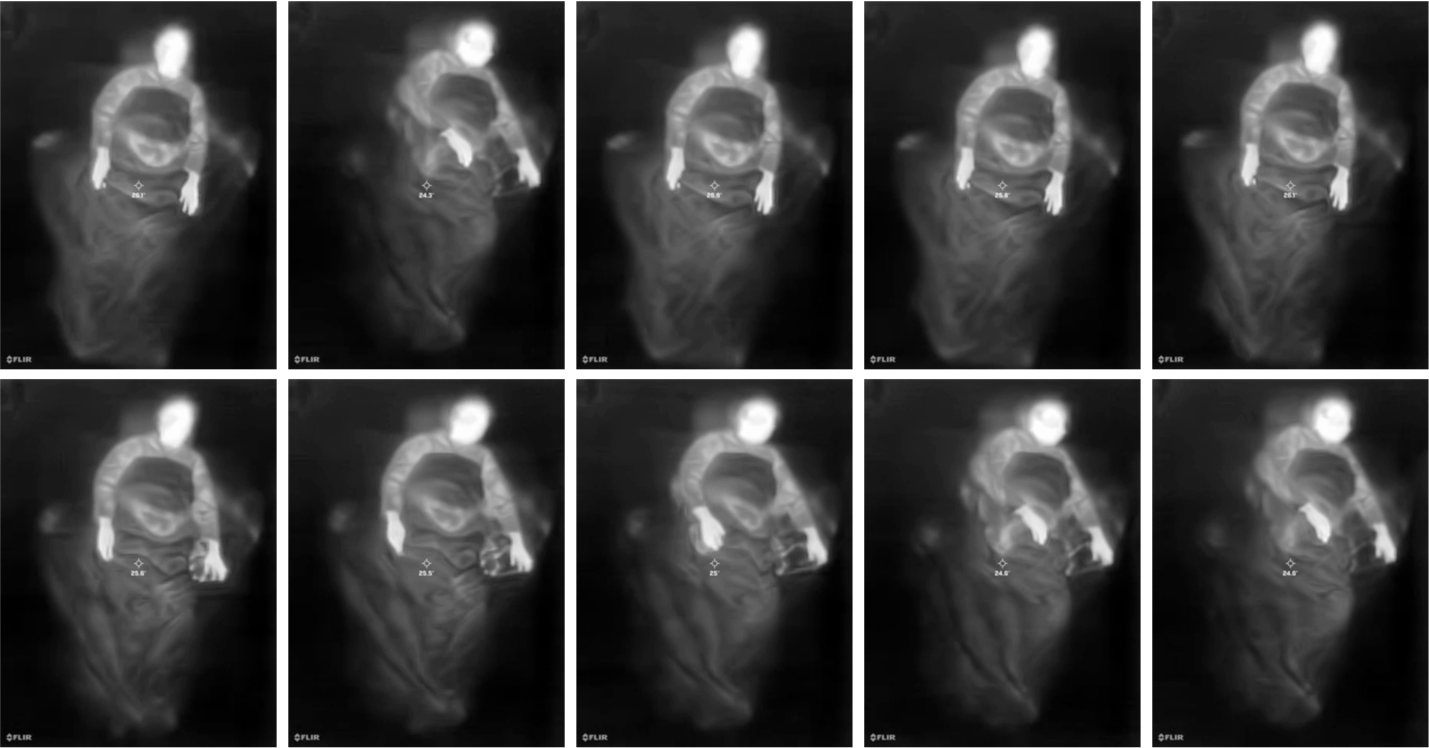


**Figure S16.** Infrared images are measured when the user is in the light sleep state.


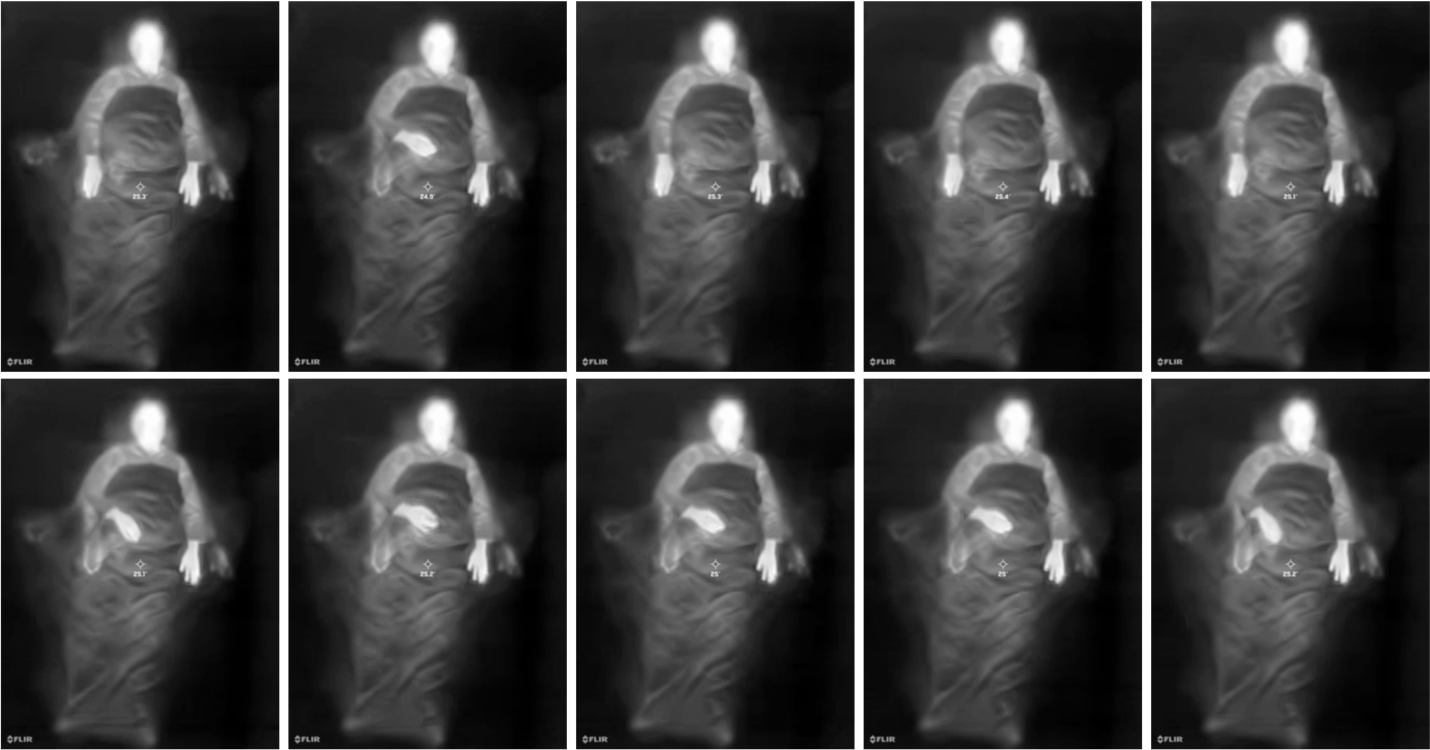


**Figure S17.** Infrared images are measured when the user is in a deep sleep state.


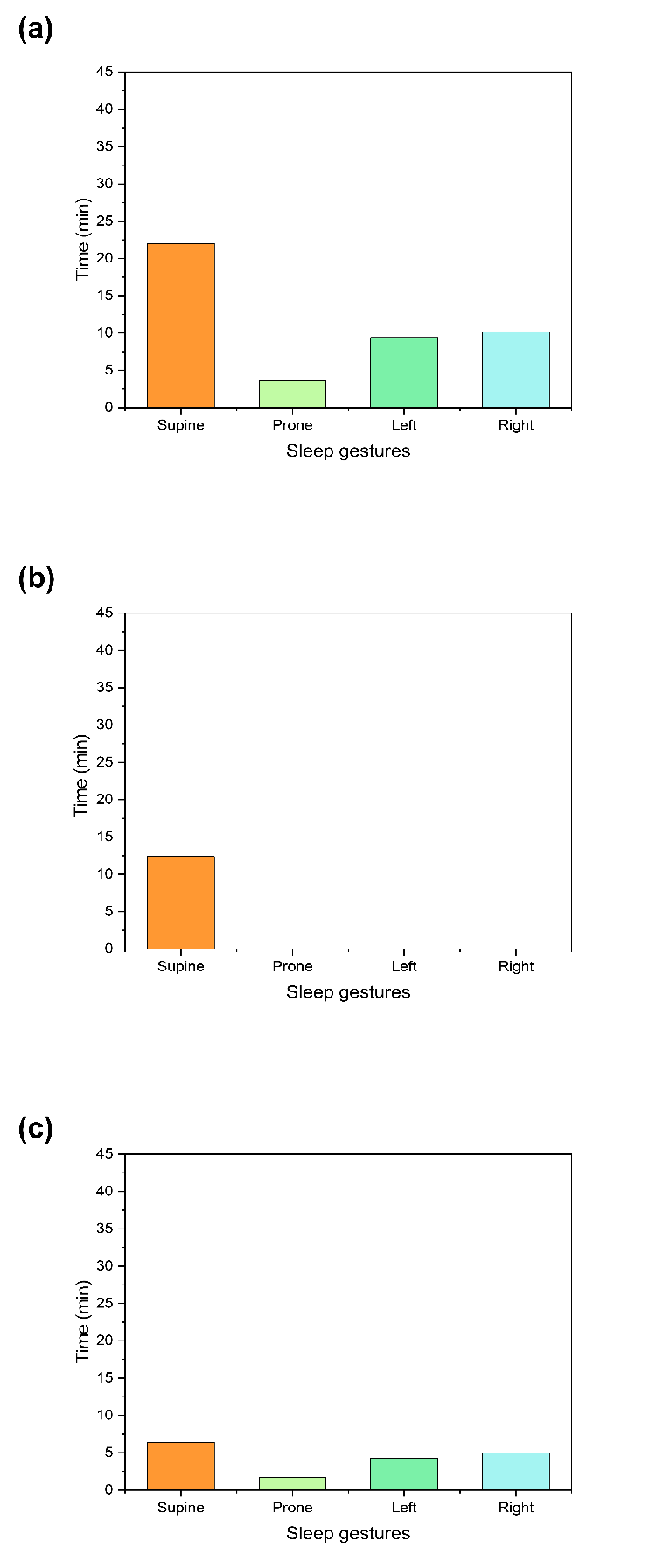


**Figure S18.** The time distribution for 4 sleep postures during different stages in one cycle. The time spent for different sleep postures during a) The awake state, b) the light sleep state, and (c) the deep sleep state.


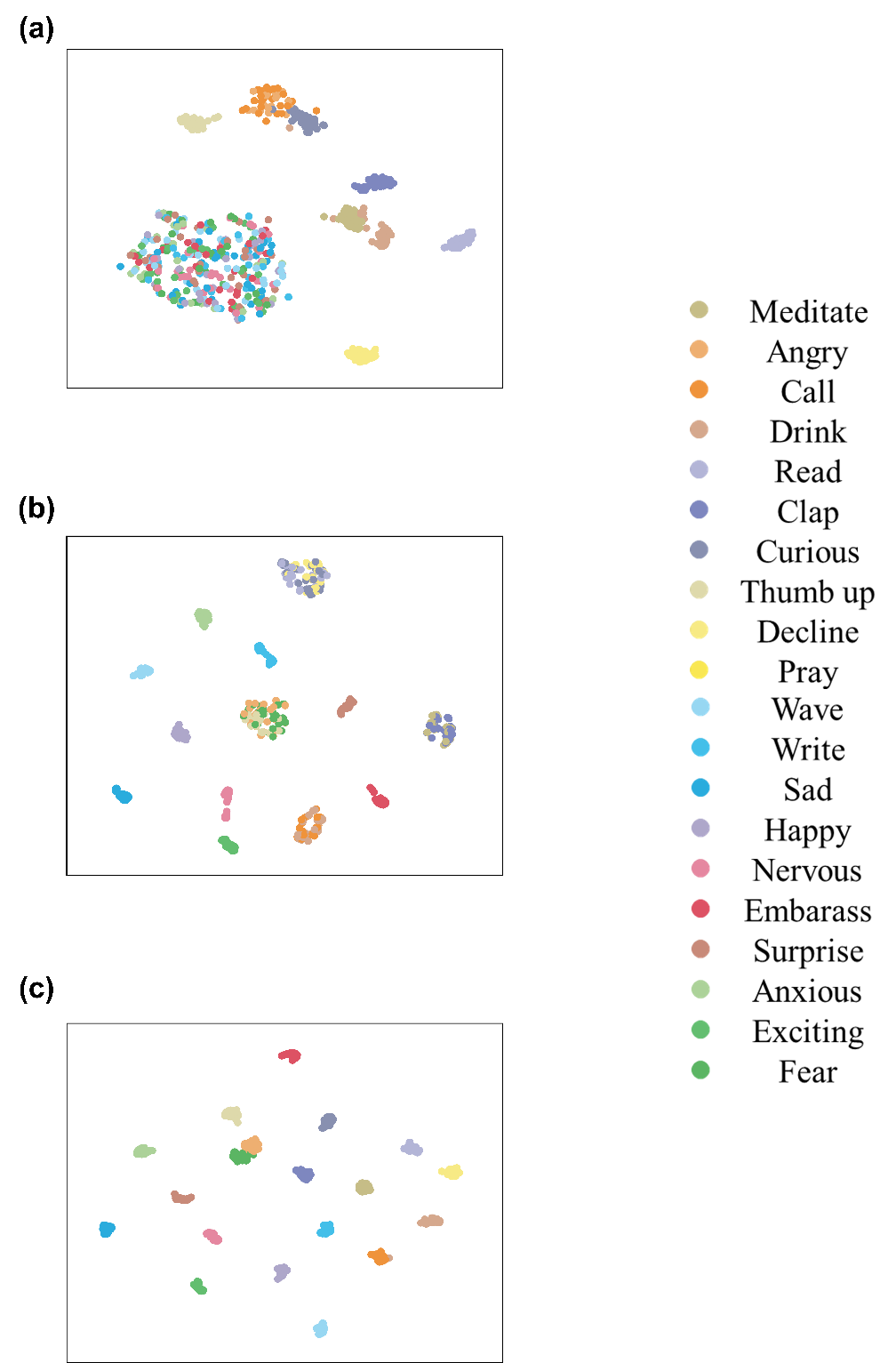


**Figure S19.** The t-SNE figures for different outputs. The t-SNE diagrams for a) triboelectric outputs only, b) infrared images only, and c) multimodal data fusion.


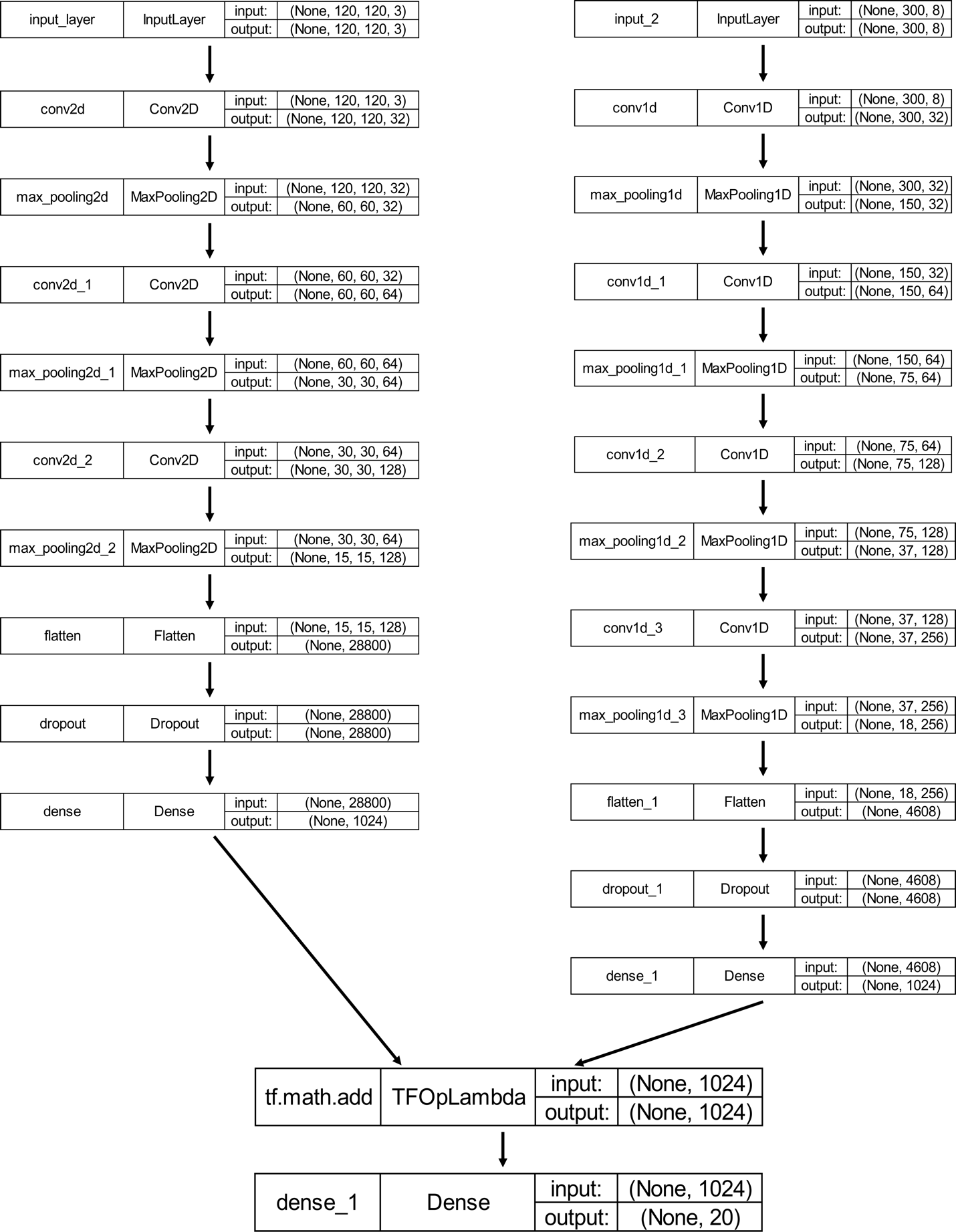


**Figure S20.** The detailed parameters of the neural network for multimodal CNN.

**Table S1.** Comparison of smart mats or mattresses for smart home applications.

| **Reference** | **Year** | **Sensing**  **Mechanism** | **Multimodality** | **Basic Signal** | **Functions** | **Accuracy** | **Demonstration** |
| --- | --- | --- | --- | --- | --- | --- | --- |
| ^[1]^ | 2020 | triboelectric | \ | Triboelectric walking signal | Identity information;  Activity status ;  Stepping position | 96% Identification (10 users  Same passing status)  89.17% Identification (4 users 12 labels) | Smart floor monitoring system |
| ^[2]^ | 2021 | triboelectric | \ | Triboelectric moving signal | Position detection ;  Identity recognition | 85.67% Identification (  20 users) | Smart building |
| ^[3]^ | 2022 | triboelectric | \ | Triboelectric moving signal | Crew identification ;  Monitoring ;  Location detection ;  People counting | 94.44% Identification (  9 users 50 epochs) | Maritime Safety |
| ^[4]^ | 2023 | triboelectric | Triboelectric - contact  Triboelectric - force | Triboelectric moving  signal  Triboelectric force signal | Position sensing;  Person identification;  Activity monitoring | 99% Identification(10 users  93% triboelectric contact channel  94% triboelectric force channel) | Yoga pose |
| ^[5]^ | 2019 | triboelectric | \ | Triboelectric vibration | Activity monitoring(lying, stand, sitting);  Respiration modes (normal, rapid, deep, hold ) | \ | Monitoring breath related diseases (OASA) |
| ^[6]^ | 2022 | triboelectric | \ | Triboelectric force | Monitoring head movement | \ | Sleep monitoring |
| ^[7]^ | 2020 |  | \ | BCG signal | Sleeping Gesture  Breath & heartbeat rate monitoring | \ | Sleep monitoring |
| ^[8]^ | 2016 | Resistive | \ | Pressure signal | Pressure mapping | \ | Tele-home care |
| ^[9]^ | 2018 | Triboelectric | \ | Triboelectric pressure signal | Sleep behavior monitoring;  Anti-falling | \ | Sleep monitoring |
| ^[10]^ | 2019 | Resistive & Capacitance | \ | Resistance of electrodes;  Capacitance of electrodes | Occupation & wetness | \ | Incontinence and occupation in elder care |
| ^[11]^ | 2009 |  | \ | Pressure sensor signal | Sit & stand classification. | \ | Smart home |
| ^[12]^ | 2020 | Resistive | \ | Pressure signal;  Heartbeat & breath signal | Detection position, motion, and breathing & heartbeat rate | 91.8% Gesture classification (6 labels)  86% REM classification (4 labels) | Sleep monitoring |
| This  Work | 2024 | Triboelectric | Triboelectric;  Thermal images | Triboelectric contact signal;  Thermal  images | Sleep posture  Recognition;  Daily behavior  detection | 96.67% Sleep posture recognition(3user, 4 posture, 12 labels);  87% behavior detection (20 behaviors) | Comprehensive sleep monitoring;  Digital twin smart home |

**Note S3.** Calculating the area ratio of each pixel by matrix computations


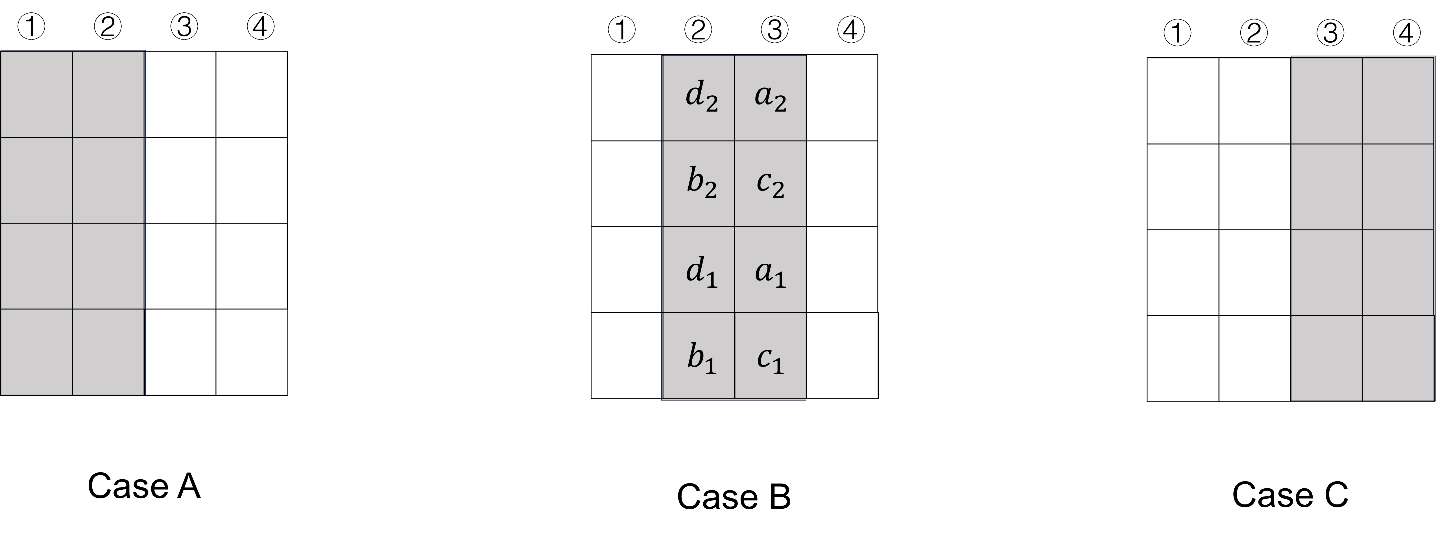


In the manuscript, after calculating from voltage integration and division, the results contain 8 channels’ area ratio output matrix $R_{1}：$

$$R_{1}=\left( A_{1} A_{2} B_{1} B_{2} C_{1} C_{2} D_{1} D_{2} \right)$$

Based on the area ratio for eight channels’ output, we use a matrix multiplication method to get the exact area ratio for each

pixel. Considering the constraints of human body dimensions, it is assumed that users can occupy no more than two columns of the mat array simultaneously. Thus, occasions of area ratio mapping can be divided into 3 cases (Case A, Case B, and Case C). We set Case B as an example to illustrate the area mapping matrix $R_{2}$ can be defined as:

$$R_{2}=\left( a_{1} a_{2} b_{1} b_{2} c_{1} c_{2} d_{1} d_{2} \right)$$

The relationship between $A_{1}$ and $A_{2}$ can be inferred from parallel connection method in Figure 2g. For channel $A_{1}$and $A_{2}$:

$$\left\{ \begin{aligned} A_{1}=0.2a_{1}+a_{2} \\ A_{2}=0.8a_{1} \end{aligned} \right.$$

Then, the equation can be expressed as:

$$\left( A_{1} A_{2} \right)\left( \begin{matrix} 0 & 1 \\ 1.25 & -0.25 \end{matrix} \right)=\left( a_{1} a_{2} \right)$$

Channels $B_{1}, B_{2}, C_{1}, C_{2}, D_{1}$ and $D_{2}$ have similar relationships. Therefore,

$$\left( A_{1} A_{2} B_{1} B_{2} C_{1} C_{2} D_{1} D_{2} \right)\left( \begin{matrix} \begin{matrix} 0 \\ 1.25 \\ 0 \end{matrix} & \begin{matrix} 1 \\ -0.25 \\ 0 \end{matrix} \\ 0 & 0 \\ \begin{matrix} 0 \\ \begin{matrix} 0 \\ 0 \\ 0 \end{matrix} \end{matrix} & \begin{matrix} 0 \\ \begin{matrix} 0 \\ 0 \\ 0 \end{matrix} \end{matrix} \end{matrix}\begin{matrix} \begin{matrix} 0 \\ 0 \\ -0.25 \end{matrix} & \begin{matrix} 0 \\ 0 \\ 1.25 \end{matrix} \\ 1 & 0 \\ \begin{matrix} 0 \\ \begin{matrix} 0 \\ 0 \\ 0 \end{matrix} \end{matrix} & \begin{matrix} 0 \\ \begin{matrix} 0 \\ 0 \\ 0 \end{matrix} \end{matrix} \end{matrix}\begin{matrix} \begin{matrix} 0 \\ 0 \\ 0 \end{matrix} & \begin{matrix} 0 \\ 0 \\ 0 \end{matrix} \\ 0 & 0 \\ \begin{matrix} -0.25 \\ \begin{matrix} 1 \\ 0 \\ 0 \end{matrix} \end{matrix} & \begin{matrix} 1.25 \\ \begin{matrix} 0 \\ 0 \\ 0 \end{matrix} \end{matrix} \end{matrix}\begin{matrix} \begin{matrix} 0 \\ 0 \\ 0 \end{matrix} & \begin{matrix} 0 \\ 0 \\ 0 \end{matrix} \\ 0 & 0 \\ \begin{matrix} 0 \\ \begin{matrix} 0 \\ 0 \\ 1.25 \end{matrix} \end{matrix} & \begin{matrix} 0 \\ \begin{matrix} 0 \\ 1 \\ -0.25 \end{matrix} \end{matrix} \end{matrix} \right)=\left( a_{1} a_{2} b_{1} b_{2} c_{1} c_{2} d_{1} d_{2} \right)$$

Finally, we can get the area mapping matrix $R_{2}$ in Case B.

**Table S2.** Summary of characters and corresponding descriptions used in the manuscript.

| **Character** | **Description** |
| --- | --- |
| E1, E2 | The 2 output channels for one triboelectric mat pixel (illustrated in **Figure S3**). In the diagram, the red electrode represents E1, and the brown electrode represents E2, with an area ratio of 1:4. Due to the contact-separation mode, the resulting voltage output will also roughly follow this 1:4 ratio. |
| a1, a2, a'1, a'2, b1, b2, b'1, b'2 | The 8 serial outputs for the separate connection of 4 mats (detailed schematic diagram in **Figure 2a**). The diagram includes 4 sets of electrodes: a1 & a2, a'1 & a'2, b'1 & b'2, and b1 & b2, depicted in blue, orange, pink, and green, respectively. The voltage ratios for the 4-pixel sets are as follows: set a1 & a2 has a voltage ratio of 0:10, set a'1 & a'2 has a ratio of 2:8, set b'1 & b'2 has a ratio of 8:2, and set b1 & b2 has a ratio of 10:0. |
| A1, A2, A'1, A'2 | The 4 serial outputs for interval parallel connection of 4 mats (detailed schematic diagram in **Figure 2d**). The diagram includes A1 & A2 in blue and A'1 & A'2 in pink. The voltage ratio sets are as follow: set A1 & A2 has voltage ratios of 0:10 and 8:2 and set A'1 & A'2 has ratios of 2:8 and 10:0. |
| A1, A2, B1, B2, C1, C2, D1, D2 | The 8 output channels of the constructed $4\times4$ pixels mat array (illustrated in **Figure 2g**). The triboelectric mat array features 4 electrode pairs (A1 & A2, B1 & B2, C1 & C2, and D1 & D2), 8 output terminals in total. Each electrode pair, marked with the same color, connects 4 mats, distinguished by its unique voltage ratio. All statements regarding “triboelectric mat array” below are specifically referring to this structure. |
| M1-M8 | 8 motions from daily life (M1='Walk', M2='Jump', M3='Lie Leftward', M4='Lie Rightward', M5='Sit', M6='Lie', M7='Run', and M8='Prone') in **Figure 2h**. |
| $\int A1dt$, $\int A2dt$ | The voltage integrals of the A1 and A2 electrodes when user is contacting the mat section without leaving it in **Figure 3b(ii)**. |
| $\Lambda$ | The contact area of the triboelectric mat array. |
| $\Gamma$ | The voltage integration when contacting the triboelectric mat array, indicating a direct proportionality to the charge transfer. |
| S1-S12 | 4 sleep postures of 3 users(S1='Supine(User1)', S2='Left(User1)', S3='Right(User1)', S4='Prone(User1)', S5='Supine(User2)', S6='Left(User2)', S7='Right(User2)', S8='Prone(User2)', S9 ='Supine(User3)', S10= 'Left(User3)', S11='Right(User3)', S12='Prone(User3)') in **Figure 4**. |
| P_TENG_ , P_IR_ | The recognition performance for solely using TENG data and solely using infrared images. |
| B1-B20 | 20 common behaviors from daily life (B1='Wave', B2='Write', B3='Call', B4='Drink', B5='Read', B6='Clap',B7='Curious', B8='Thumb up', B9='Decline', B10='Pray', B11='Sad', B12='Happy', B13='Nervous', B14='Anxious', B15='Fear', B16='Angry', B17='Contemplate', B18='Embarrass', B19='Exciting', and B20='Surprise') in **Figure 6**. |

**Table S3.** Summary of abbreviations and full term used in the manuscript.

| **Abbreviation** | **Full Term** |
| --- | --- |
| AI | artificial intelligence |
| AR | augmented reality |
| CNN | convolutional neural network |
| DL | deep learning |
| IDEs | interdigital electrodes |
| IoT | Internet of Things |
| MEMS | microelectromechanical system |
| PENGs | piezoelectric nanogenerators |
| PET | polyethylene terephthalate |
| PTFE | polytetrafluoroethylene |
| PVC | polyvinyl chloride |
| TENGs | triboelectric nanogenerators |
| TMMSS | triboelectric mat multimodal sensing system |
| TPE | thermoplastic elastomer |
| t-SNE | t- distributed stochastic neighbor embedding |
| VR | virtual reality |

**Reference:**

[1] Q. Shi, Z. Zhang, T. He, Z. Sun, B. Wang, Y. Feng, X. Shan, B. Salam, C. Lee, *Nat. Commun.* **2020**, *11*, DOI 10.1038/s41467-020-18471-z.

[2] Q. Shi, Z. Zhang, Y. Yang, X. Shan, B. Salam, C. Lee, *ACS Nano* **2021**, *15*, DOI 10.1021/acsnano.1c07579.

[3] Y. Wang, Z. Hu, J. Wang, X. Liu, Q. Shi, Y. Wang, L. Qiao, Y. Li, H. Yang, J. Liu, L. Zhou, Z. Yang, C. Lee, M. Xu, *ACS Appl. Mater. Interfaces* **2022**, *14*, DOI 10.1021/acsami.2c05734.

[4] Y. Yang, Q. Shi, Z. Zhang, X. Shan, B. Salam, C. Lee, *InfoMat* **2023**, *5*, DOI 10.1002/inf2.12360.

[5] H. Zhang, J. Zhang, Z. Hu, L. Quan, L. Shi, J. Chen, W. Xuan, Z. Zhang, S. Dong, J. Luo, *Nano Energy* **2019**, *59*, 75.

[6] H. Kou, H. Wang, R. Cheng, Y. Liao, X. Shi, J. Luo, D. Li, Z. L. Wang, *ACS Appl. Mater. Interfaces* **2022**, *14*, 23998.

[7] Z. Zhou, S. Padgett, Z. Cai, G. Conta, Y. Wu, Q. He, S. Zhang, C. Sun, J. Liu, E. Fan, K. Meng, Z. Lin, C. Uy, J. Yang, J. Chen, *Biosens. Bioelectron.* **2020**, *155*, DOI 10.1016/j.bios.2020.112064.

[8] J. F. Saenz-Cogollo, M. Pau, B. Fraboni, A. Bonfiglio, *Sensors (Switzerland)* **2016**, *16*, 1.

[9] Z. Lin, J. Yang, X. Li, Y. Wu, W. Wei, J. Liu, J. Chen, J. Yang, *Adv. Funct. Mater.* **2018**, *28*, 1.

[10] M. Fischer, M. Renzler, T. Ussmueller, *IEEE Access* **2019**, *7*, 118498.

[11] A. Arcelus, C. L. Herry, R. A. Goubran, F. Knoefel, H. Sveistrup, M. Bilodeau, H. Sveistrup, M. Bilodeau, *IEEE Trans. Biomed. Eng.* **2009**, *56*, 2485.

[12] M. Laurino, L. Arcarisi, N. Carbonaro, A. Gemignani, D. Menicucci, A. Tognetti, *IEEE Access* **2020**, *8*, 45664.
